# Pharmacokinetics of Oral Formulations of Gepotidacin (GSK2140944), a Triazaacenaphthylene Bacterial Type II Topoisomerase Inhibitor, in Healthy Adult and Adolescent Participants

**DOI:** 10.1128/AAC.01263-21

**Published:** 2022-01-18

**Authors:** Aline Barth, Mohammad Hossain, Darin B. Brimhall, Caroline R. Perry, Courtney A. Tiffany, Sherry Xu, Etienne F. Dumont

**Affiliations:** a GlaxoSmithKline, Collegeville, Pennsylvania, USA; b PPD, Las Vegas, Nevada, USA

**Keywords:** gepotidacin, pharmacokinetics, relative bioavailability, adolescents, safety

## Abstract

Gepotidacin is a novel, first-in-class triazaacenaphthylene antibiotic that may provide a new treatment option for antibiotic-resistant pathogens. Two pharmacokinetic evaluations of oral gepotidacin are presented: a relative bioavailability study that guided formulation development, followed by an adult and adolescent study of the final formulation. In the relative bioavailability study, after gepotidacin administration to 26 healthy adults as free-base roller-compacted (RC) tablets, free-base high-shear wet granulation (HSWG) tablets, and mesylate salt reference capsules, the RC tablet exposure ratios and 90% confidence intervals (CIs) were within the 0.80-to-1.25 confidence bounds; however, the HSWG tablet maximum observed concentration (*C*_max_) was higher than the reference (ratio, 1.15; 90% CI, 1.0113, 1.3047). In the healthy adult (*n* = 16) and adolescent (*n* = 17) study, a gepotidacin mesylate salt tablet was evaluated as a 1,500-mg single dose or 2 doses administered 6 or 12 h apart (6,000 mg total), or placebo was administered. The single-dose mean *C*_max_ was ∼27% higher in adolescents than in adults, and area under the concentration-time curve (AUC) values were comparable in both populations. After 2 doses were administered, the mean *C*_max_ values were similar for both age groups, and the mean AUC was ∼35% higher in adolescents than in adults. Concentrations increased proportionally with dose. Safety-risk profiles were similar for both age groups. Across studies, the most common adverse events were gastrointestinal. Overall, the pharmacokinetics of the final gepotidacin mesylate salt tablet have been well characterized, enrollment of adolescents into the pivotal trials is supported, and dosing intervals were determined that should provide adequate exposures for microbiological efficacy. (This study has been registered at ClinicalTrials.gov under identifiers NCT02853435 and NCT04079790.)

## TEXT

Gepotidacin is a novel, first-in-class triazaacenaphthylene antibiotic that inhibits bacterial DNA replication by a distinct mechanism of action (1, 2), which confers activity against most strains of Escherichia coli, Staphylococcus saprophyticus, and Neisseria gonorrhoeae, including those resistant to current antibiotics (3–7). Phase 2 clinical trials indicated that gepotidacin may be a viable treatment for urogenital gonorrhea, uncomplicated urinary tract infection (UTI), and acute bacterial skin and skin structure infections (8–10). Phase 3 confirmatory studies are currently in progress to support urogenital gonorrhea (ClinicalTrials.gov identifier NCT04010539) and uncomplicated UTI (ClinicalTrials.gov identifiers NCT04020341 and NCT04187144) indications.

Throughout phase 1 and 2 clinical development, the pharmacokinetics (PK) of gepotidacin have been well defined in healthy adult participants (8–15) and in participants with renal or hepatic impairment (16, 17). Gepotidacin formulations used in the PK evaluations included mesylate salt for an intravenous solution, capsules, tablets, and the free base for capsules and tablets. As clinical evaluation progressed, the mesylate salt oral tablet formulation was selected for potential commercialization. Here, we present data from 2 clinical studies that further evaluated gepotidacin PK and formulations. Results from a relative bioavailability study that compared 2 different free-base tablet formulations with a reference mesylate salt capsule formulation are presented. In addition, results are presented from a second clinical study with a mesylate salt tablet formulation. This study defined the PK of the final formulation for phase 3 evaluation, assessed the safety and systemic exposure of gepotidacin in a younger population to support the inclusion of adolescents ≥12 years of age in the phase 3 studies, and evaluated the optimum dosing time window for the second dose in the treatment of urogenital gonorrhea.

## RESULTS

### Relative bioavailability study.

Twenty-six healthy adult participants were enrolled in this open-label study and received single oral doses of gepotidacin at 1,500 mg as two 750-mg roller-compacted (RC) free-base tablets, two 750-mg free-base high-shear wet granulation (HSWG) tablets, and three 500-mg mesylate salt reference capsules under fasted conditions in a randomized order separated by a washout of 3 days. All participants completed the study. The population consisted of 21 males and 5 females with a mean (range) body weight of 77.98 kg (54.6 to 115.0 kg) (Table 1).

**TABLE 1:** Baseline demographics across clinical studies

Demographic	Value for group						
	Adult relative BA^*a*^ study of gepotidacin (*n* = 26)	Adult and adolescent study					
		Adults			Adolescents		
		Gepotidacin (*n* = 14)	Placebo (*n* = 2)	Total (*n* = 16)	Gepotidacin (*n* = 14)	Placebo (*n* = 3)	Total (*n* = 17)^*b*^
Mean age (yrs) (SD)	39.5 (10.64)	43.7 (9.65)	58.5 (7.78)	45.6 (10.50)	14.4 (2.10)	14.3 (0.58)	14.4 (1.91)
Sex [no. (%) of participants]							
Female	5 (19.2)	6 (43)	1 (50)	7 (44)	4 (29)	1 (33)	5 (29)
Male	21 (80.8)	8 (57)	1 (50)	9 (56)	10 (71)	2 (67)	12 (71)
Mean body mass index (kg/m^2^) (SD)	26.5 (3.2)	26.6 (2.7)	27.4 (1.1)	26.7 (2.5)	24.2 (4.6)	21.9 (3.1)	23.8 (4.4)
Mean ht (cm) (SD)	171.2 (9.6)	172.1 (10.8)	167.2 (4.5)	171.5 (10.3)	163.0 (7.8)	166.5 (17.2)	163.6 (9.4)
Mean wt (kg) (SD)	78.0 (14.5)	79.4 (14.0)	76.6 (7.1)	79.1 (13.2)	64.1 (12.1)	62.1 (19.0)	63.7 (12.8)
No. (%) of participants of ethnicity							
Hispanic or Latino	13 (50.0)	2 (14)	1 (50)	3 (19)	8 (57)	1 (33)	9 (53)
Not Hispanic or Latino	13 (50.0)	12 (86)	1 (50)	13 (81)	6 (43)	2 (67)	8 (47)
No. (%) of participants of race							
Asian—Central/South Asian heritage	0	1 (7)	0	1 (6)	1 (7)	0	1 (6)
Black or African American	10 (38)	6 (43)	0	6 (38)	6 (43)	1 (33)	7 (41)
Multiple	0	0	0	0	2 (14)	0	2 (12)
White—Arabic/North African heritage	0	1 (7)	0	1 (6)	0	0	0
White—white/Caucasian/European heritage	16 (62)	6 (43)	2 (100)	8 (50)	5 (36)	2 (67)	7 (41)

a BA, relative bioavailability.

b A total of 18 adolescent participants were enrolled. One adolescent randomly assigned to active gepotidacin was unable to swallow the tablets and thus was not included in the safety population.

### (i) Pharmacokinetics.

**FIG 1:**
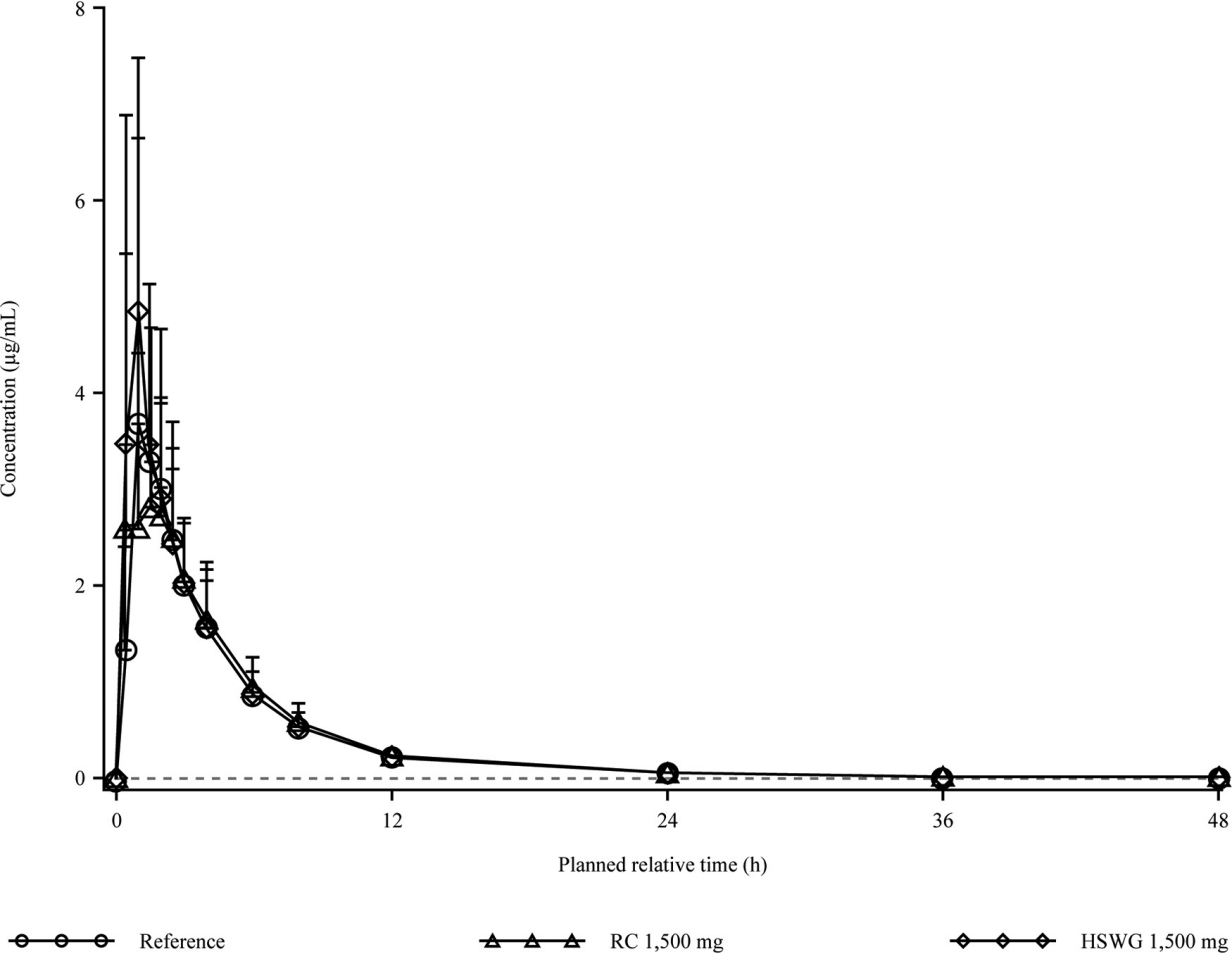
Arithmetic mean (standard deviation [SD]) plasma gepotidacin concentration-time plot by treatment on a linear scale (*n* = 26 per treatment group) for the relative bioavailability study. Note that the lower limit of quantification is 0.01 μg/mL, represented by the dashed line. HSWG, high-shear wet granulation; RC, roller compacted.

The 2 free-base RC and HSWG tablet formulations and the reference mesylate salt capsule formulation of gepotidacin were rapidly absorbed in plasma under fasted conditions, with similar time to reach the maximum observed concentration (*T*_max_) and terminal elimination half-life (*t*_1/2_) values (Fig. 1 and Table 2). Intersubject variabilities across the 3 formulations were low for area under the concentration-time curve (AUC) from time zero to infinity (AUC_0–∞_) (range, 23.0% to 25.7%) and moderate for maximum observed concentration (*C*_max_) (range, 43.6% to 46.8%) values. The 90% confidence intervals (CIs) of the ratio of the free-base RC tablet to the mesylate salt capsule were within the 0.80-to-1.25 bioequivalence confidence bounds (18) for the AUC from time zero to the last quantifiable concentration (AUC_0–_*_t_*) (0.9792, 1.1062), AUC_0–∞_ (0.9809, 1.1063), and *C*_max_ (0.8440, 1.0888). Compared to the reference capsule, the free-base HSWG tablet met the 90% CI criteria for AUC_0–_*_t_* (1.0479, 1.1838) and AUC_0–∞_ (1.0459, 1.1797); however, the upper CI for *C*_max_ (1.0113, 1.3047) did not meet the criteria (see Table S1 in the supplemental material).

**TABLE 2:** Summary of oral gepotidacin plasma PK parameters in healthy participants across clinical studies^*a*^

PK parameter^*b*^	Value for group										
	Relative bioavailability study (fasted)			Adult and adolescent study (administered with food)							
	Adults, FB RC tablet, single dose, 1,500 mg (*n* = 26)	Adults, FB HSWG tablet, single dose, 1,500 mg (*n* = 26)	Adults, MS capsule, single dose, 1,500 mg (*n* = 26)	Adults, MS tablet, single dose, 1,500 mg (*n* = 14)	Adolescents, MS tablet, single dose, 1,500 mg (*n* = 13)	Adults, MS tablet, 2 doses 12 h apart		Adults, MS tablet, 2 doses 6 h apart		Adolescents, MS tablet, 2 doses 6 h apart	
						Dose 1,^*c*^ 3,000 mg (*n* = 13)	Dose 2,^*c*^ 3,000 mg	Dose 1,^*c*^ 3,000 mg (*n* = 13)	Dose 2,^*c*^ 3,000 mg	Dose 1,^*c*^ 3,000 mg (*n* = 12)	Dose 2,^*c*^ 3,000 mg
*C*_max_ (μg/mL)	4.49 (43.6)	5.35 (46.8)	4.65 (43.8)	3.57 (38.1)	4.52 (29.5)	9.94 (24.2)	11.0 (28.1)	8.42 (41.8)	13.0 (28.6)	10.9 (26.8)	14.3 (29.5)
*T*_max_ (h)	1.50 (0.50, 4.00)	1.00 (0.50, 3.00)	1.50 (1.00, 4.00)	3.00 (0.50, 6.00)	3.00 (1.50, 6.00)	2.00 (1.00, 4.00)	1.57 (1.00, 4.00)	2.63 (0.50, 5.42)	1.50 (1.00, 3.28)	2.75 (1.00, 4.00)	1.50 (1.00, 3.00)
AUC_0–_*_t_* (μg · h/mL)	17.1 (23.4)	18.3 (24.1)	16.4 (26.2)	19.7 (17.6)	23.3 (21.6)	91.2 (22.6)	—^*d*^	87.1 (26.3)	—	116 (23.8)	—
AUC_0–∞_ (μg · h/mL)	17.5 (23.0)	18.6 (24.0)	16.7 (25.7)	20.2 (16.8)	23.8 (20.9)	—	—	—	—	—	—
AUC_0–24_ (μg · h/mL)	—	—	—	18.7 (19.5)	22.1 (22.6)	83.5 (22.7)	—	82.4 (27.3)	—	111 (23.8)	—
AUC_0–48_ (μg · h/mL)	—	—	—	19.7 (17.6)	23.3 (21.6)	90.5 (22.7)	—	86.5 (26.4)	—	116 (23.8)	—
AUC_0–τ_ (μg · h/mL)	—	—	—	—	—	38.2 (24.3)	44.4 (22.8)	24.1 (33.6)	40.1 (29.5)	32.4 (22.0)	53.9 (26.7)
*T*_lag_ (h)	0.00 (0.00, 0.50)	0.00 (0.00, 0.50)	0.00 (0.00, 0.00)	0.00 (0.00, 1.50)	0.50 (0.00, 1.50)	0.00 (0.00, 0.00)		0.00 (0.00, 0.00)	—	0.00 (0.00, 0.50)	—
*t*_1/2_ (h)	10.24 (15.2)	10.24 (12.1)	10.29 (13.7)	11.5 (36.2)	13.0 (16.6)	—	11.0 (27.3)	—	12.0 (14.6)	—	6.98 (19.7)
CL/*F* (L/h)	—	—	—	74.4 (16.8)	63.1 (20.9)	65.3 (22.3)	—	68.4 (26.2)	—	51.6 (23.6)	—
*V_z_*/*F* (L)	—	—	—	1,239 (39.3)	1,181 (32.9)	1,033 (41.9)	—	1,186 (30.8)	—	520 (36.6)	—
*F*_rel_	1.05 (22.3)	1.11 (16.5)	—	—	—	—	—	—	—	—	—
*R*_o_ *C*_max_	—	—	—	—	—	—	1.11 (30.6)	—	1.54 (25.9)	—	1.32 (32.8)
*R*_o_ AUC_0–τ_	—	—	—	—	—	—	1.16 (12.7)	—	1.67 (25.9)	—	1.66 (18.3)

a Values are presented as geometric means (%CVb [percent between-participant geometric coefficients of variation]), except for *T*_max_, which is presented as medians (minima, maxima). The gepotidacin strength of all capsules and tablets administered was 750 mg; multiple capsules/tablets were administered to provide each required dose. FB, free base; HSWG, high-shear wet granulation; MS, mesylate salt; *n*, number of participants with evaluable PK parameter data; RC, roller compacted.

b AUC, area under the concentration-time curve from time zero to the time point indicated; CL/*F*, apparent clearance; *C*_max_, maximum observed concentration; *F*_rel_, relative bioavailability of drug; *R*_o_, accumulation ratio based on dose 2/dose 1; τ, 6- or 12-h dosing interval; *T*_lag_, lag time before observation of drug concentrations; *T*_max_, time of occurrence of *C*_max_; *V_z_*/*F*, apparent volume of distribution.

c The AUC_0–τ_, *C*_max_, and *T*_max_ PK parameters were estimated separately by dose when 2 doses were administered. Otherwise, parameters were estimated using the full profile; the *t*_1/2_ was based on plasma concentrations after the second dose.

d Dashes indicate where data were not determined.

**FIG 2:**
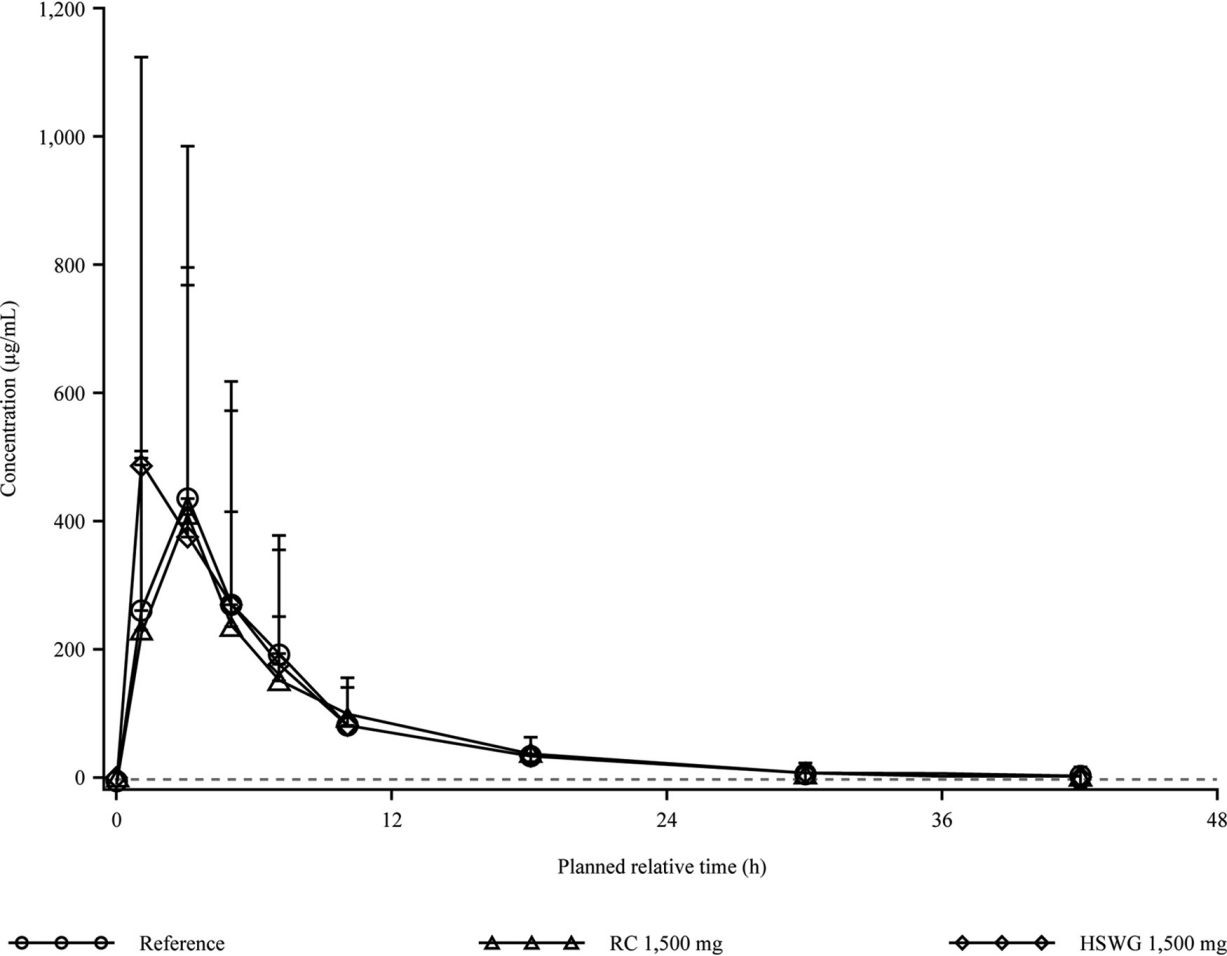
Arithmetic mean (SD) urine gepotidacin concentration-time plot by treatment on a linear scale (*n* = 26 per treatment group) for the relative bioavailability study. Note that the lower limit of quantification is 1 μg/mL, represented by the dashed line. As urine collection is representative of a time interval and not a single time point, the average concentrations at the midpoint of the collection intervals are used for plotting purposes only. HSWG, high-shear wet granulation; RC, roller compacted.

For all 3 formulations evaluated, urine concentrations were measurable over the entire 48-h collection interval, with similar renal clearance (CL_R_) and percentage of the given dose excreted in urine (*fe*%) values (Fig. 2 and Table 3). As urine collection is representative of a time interval and not a single time point, the average concentrations at the midpoint of the collection intervals are used for plotting purposes in Fig. 2, while Table 3 details the specific time point from time zero to the time point indicated.

**TABLE 3:** Summary of oral gepotidacin urine PK parameters in healthy participants across clinical studies^*a*^

PK parameter^*b*^	Value for group							
	Adult relative bioavailability study (fasted)			Adult and adolescent study (administered with food)				
	FB RC tablet, 1,500-mg single dose (*n* = 26)	FB HSWG tablet, 1,500-mg single dose (*n* = 26)	MS capsule, 1,500-mg single dose (*n* = 26)	Adults, MS tablet, 1,500-mg single dose (*n* = 14)	Adolescents, MS tablet, 1,500-mg single dose (*n* = 13)	Adults, MS tablet, 2 × 3,000-mg doses 12 h apart (*n* = 13)	Adults, MS tablet, 2 × 3,000-mg doses 6 h apart (*n* = 13)	Adolescents, MS tablet, 2 × 3,000-mg doses 6 h apart (*n* = 12)
AUC_0–τ_ (μg · h/mL)	—^*g*^	—	—	—	—	6,300 (61.2)	3,040 (86.7)	4,520 (74.5)
AUC_0–12_ (μg · h/mL)^*c*^	1,948 (61.2)	2,156 (74.8)	1,904 (82.7)	—	—	—	—	—
AUC_0–24_ (μg · h/mL)^*c*^^,^^*d*^	2,464 (56.3)	2,599 (69.0)	2,373 (74.9)	2,750 (69.4)	3,660 (87.2)	15,000 (62.4)	11,000 (69.9)	19,200 (63.1)
AUC_0–48_ (μg · h/mL)^*c*^^,^^*d*^	2,725 (54.4)	2,768 (64.0)	2,597 (69.5)	2,980 (67.0)	4,070 (84.7)	16,400 (62.4)	12,000 (68.9)	21,400 (63.4)
8–12-h concn (μg/mL)	—	—	—	147 (153)	155 (60.9)	—	—	—
CL_R_ (L/h)^*e*^	16.7 (19.6)	15.5 (22.3)	16.8 (21.7)	16.4 (19.6)	15.1 (25.9)	15.8 (13.0)	14.3 (33.3)	14.5 (26.4)
CL_R_/body wt (L/h/kg)	0.218 (18.9)	0.202 (25.3)	0.218 (20.1)	—	—	—	—	—
*fe*%^*f*^	19.1 (22.2)	19.0 (30.0)	18.3 (28.6)	21.5 (19.7)	23.4 (25.5)	24.0 (15.3)	20.7 (31.7)	27.9 (25.3)
*Ae* total (mg)	287 (22.2)	285 (30.0)	275 (28.6)	322 (19.7)	352 (25.5)	1,440 (15.3)	1,240 (31.7)	1,670 (25.3)

a Values are presented as geometric means (%CVb [percent between-participant geometric coefficients of variation]), except for concentrations, which are presented as arithmetic means (%CV). The gepotidacin strength of all capsules and tablets administered was 750 mg; multiple capsules/tablets were administered to provide each required dose. FB, free base; HSWG, high-shear wet granulation; MS, mesylate salt; *n*, number of participants with evaluable PK parameter data; RC, roller compacted.

b *Ae* total, total unchanged drug; AUC, area under the concentration-time curve from time zero to the time point indicated; CL_R_, renal clearance; *fe*%, percentage of drug excreted; τ, 6- or 12-h dosing interval.

c The minimum urine AUC_0–12_, AUC_0–24_, and AUC_0–48_ values in the relative bioavailability study were as follows: 807, 1,132, and 1,382 μg · h/mL, respectively, for the FB RC tablet; 700, 865, and 977 μg · h/mL, respectively, for the FB HSWG tablet; and 664, 995, and 1,158 μg · h/mL, respectively, for the MS capsule.

d The minimum urine AUC_0–24_ and AUC_0–48_ values in the adult and adolescent study were as follows: 1,230 and 1,300 μg · h/mL, respectively, in adults and 618 and 659 μg · h/mL, respectively, in adolescents after a 1,500-mg single dose; 6,920 and 7,430 μg · h/mL in adults after two 3,000-mg doses 12 h apart; and 4,410 and 4,630 μg · h/mL, respectively, in adults and 5,840 and 6,240 μg · h/mL, respectively, in adolescents after two 3,000-mg doses 6 h apart.

e The CL_R_ was calculated as *Ae* total/AUC_0–_*_t_*.

f The *fe*% and *Ae* total account for both doses of gepotidacin when 2 doses were administered.

g Dashes indicate where data were not determined.

### (ii) Safety and tolerability.

Adverse events (AEs) were experienced by 50% of the participants (Table 4). The incidences of AEs were comparable when participants received HSWG tablets (35%) and mesylate salt capsules (31%) and were the lowest for the RC tablets (19%). Half of the participants experienced gastrointestinal AEs; the overall incidence was >10% for the AEs of diarrhea, abdominal pain, nausea, and flatulence.

**TABLE 4:** Summary of adverse events for oral gepotidacin mesylate salt reference capsules and free-base roller-compacted and high-shear wet granulation tablets in healthy adult participants in the relative bioavailability study^*a*^

System organ class, preferred term	No. (%) of participants in gepotidacin 1,500-mg single-dose group			
	MS reference capsules (*n* = 26)	FB RC tablets (*n* = 26)	FB HSWG tablets (*n* = 26)	Total (*n* = 26)
Any adverse event	8 (31)	5 (19)	9 (35)	13 (50)
Gastrointestinal disorders	7 (27)	5 (19)	9 (35)	13 (50)
Diarrhea	6 (23)	5 (19)	6 (23)	10 (39)
Abdominal pain	2 (8)	2 (8)	5 (19)	6 (23)
Nausea	1 (4)	1 (4)	2 (8)	4 (15)
Flatulence	1 (4)	2 (8)	0	3 (12)
Abdominal distension	1 (4)	1 (4)	0	2 (8)
Dry mouth	1 (4)	0	0	1 (4)
Dyspepsia	0	0	1 (4)	1 (4)
Abnormal gastrointestinal sounds	0	0	1 (4)	1 (4)
Oral paresthesia	0	0	1 (4)	1 (4)
Salivary hypersecretion	0	0	1 (4)	1 (4)
General disorders and administration site conditions	1 (4)	1 (4)	0	2 (8)
Feeling cold	0	1 (4)	0	1 (4)
Medical device site dermatitis	1 (4)	0	0	1 (4)
Nervous system disorders	1 (4)	0	1 (4)	2 (8)
Dizziness	1 (4)	0	0	1 (4)
Headache	0	0	1 (4)	1 (4)
Eye disorders	0	0	1 (4)	1 (4)
Vision blurred	0	0	1 (4)	1 (4)
Infections and infestations	1 (4)	0	0	1 (4)
Epididymitis	1 (4)	0	0	1 (4)
Musculoskeletal and connective tissue disorders	1 (4)	0	0	1 (4)
Musculoskeletal pain	1 (4)	0	0	1 (4)

a The gepotidacin strength of all capsules and tablets administered was 750 mg; multiple capsules/tablets were administered to provide each required dose. FB, free base; HSWG, high-shear wet granulation; MS, mesylate salt; RC, roller compacted.

Four of 26 participants (15%) experienced AEs of moderate severity; all other AEs reported were of mild severity, and no severe AEs were reported. One participant reported a moderate AE of headache that was related to the HSWG tablets. Three participants reported moderate AEs of diarrhea, 1 related to the reference capsules and 2 related to the RC tablets. In 1 of these participants, gepotidacin administration was delayed in the next study period by approximately 2 h due to AEs of abdominal distension, flatulence, and diarrhea; however, the participant received the planned dose and completed the study without further delays or interruptions. There were no deaths, serious AEs, or AEs that led to discontinuation.

Clostridium difficile testing was performed for 2 participants; nucleic acid amplification test results and toxin A/B assay results were all negative. No clinically significant trends were observed in laboratory values, electrocardiogram (ECG) parameters, or vital sign measurements.

### Adult and adolescent study.

**FIG 3:**
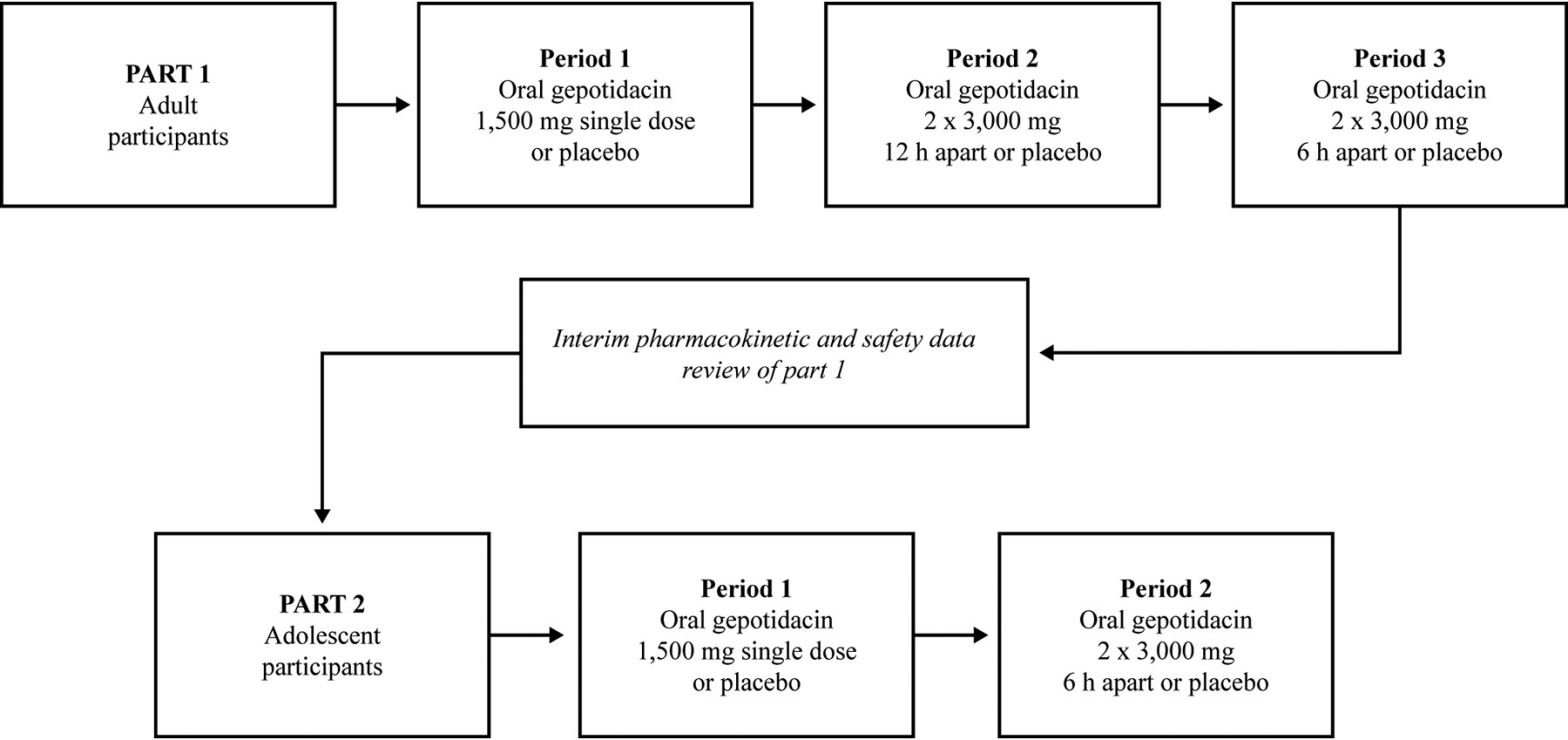
Adult and adolescent study design. Note that participants were randomly assigned in a 13:3 (part 1) or 14:3 (part 2) ratio to active:placebo for the duration of all study periods. The dosing interval for part 2 period 2 was determined after the interim data review of part 1 adult data.

The adult and adolescent study was a double-blind, 2-part, sequential PK evaluation (Fig. 3) of the 750-mg gepotidacin mesylate salt commercial tablet formulation in adults and adolescents. Multiple tablets were administered to provide each required dose. All doses were administered with food.

In part 1, 14 healthy adult participants (≥18 to ≤64 years of age, inclusive) were randomly assigned to receive active oral doses of gepotidacin at 1,500 mg as a single dose, 3,000 mg given 12 h apart (6,000-mg total dose), and 3,000 mg given 6 h apart (6,000-mg total dose) in a fixed order separated by a washout of 3 days. Two adult participants were randomly assigned to receive matching placebo tablets under the same conditions. Of the 16 enrolled participants, 1 did not complete the study due to a family emergency. The adult safety population (*n* = 16) consisted of 9 males and 7 females with a mean (range) body weight of 79.08 kg (47.0 to 103.2 kg) and a mean (range) age of 45.6 years (27 to 64 years) (Table 1).

In part 2, 15 healthy adolescent participants (≥12 to <18 years of age, inclusive) were randomly assigned to receive active oral doses of gepotidacin at 1,500 mg as a single dose and 3,000 mg given 6 h apart (6,000-mg total dose) in a fixed order separated by a washout of 7 days. Three adolescent participants were randomly assigned to receive matching placebo tablets under the same conditions. The 6-h dose schedule for the adolescent participants was determined based on data review of adult safety and PK data from part 1. Of the 18 enrolled participants, 1 did not complete the study due to difficult venipunctures, 1 was able to swallow only the first tablet of the first dose and was withdrawn, and 1 was unable to swallow any tablets. Thus, 1 adolescent participant did not receive any study drug. The adolescent safety population (*n* = 17) consisted of 12 males and 5 females with a mean (range) body weight of 63.71 kg (40.5 to 88.1 kg) and a mean (range) age of 14.4 years (12 to 17 years) (Table 1). The mean body weight in adolescents was approximately 15 kg lower than that in adults.

### (i) Adult pharmacokinetics.

**FIG 4:**
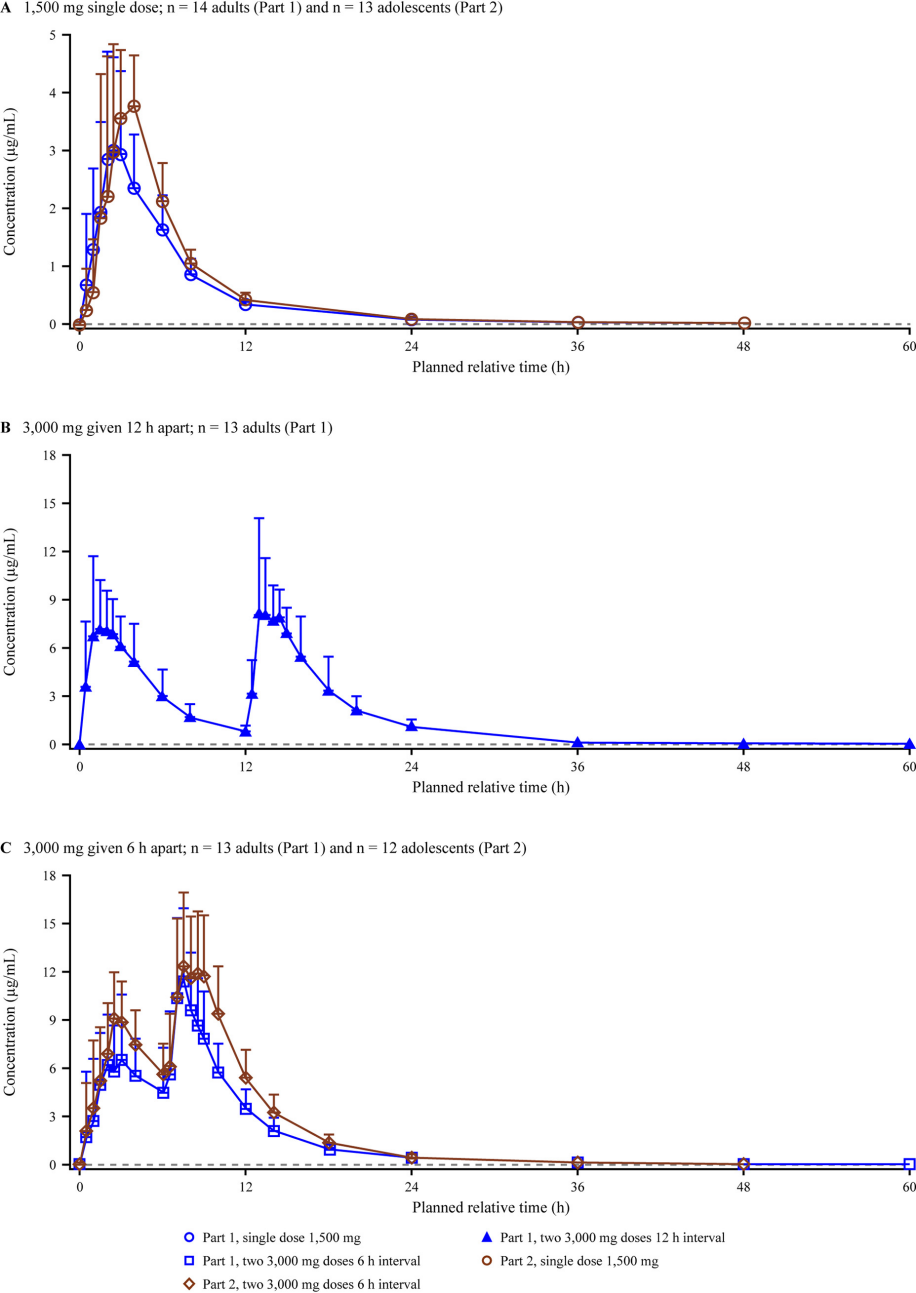
Arithmetic mean (SD) gepotidacin plasma concentration-time plots on a linear scale for the adult and adolescent study. Note that study part 1 included adults and study part 2 included adolescents. The lower limit of quantification is 0.01 μg/mL, represented by the dashed line.

After a single dose of gepotidacin, plasma concentrations peaked at 3.00 h (Fig. 4). When 2 doses of gepotidacin were administered 12 or 6 h apart, 2 plasma concentration peaks were observed approximately 2.25 h and 1.5 h after the first and second doses, respectively. Maximum plasma concentrations were approximately 2.5-fold higher after each first dose of 3,000 mg than with the 1,500-mg single dose (Table 2). Mean AUC_0–_*_t_* values, which were based on the full concentration-time profile, were approximately 4.4- to 4.6-fold higher after two 3,000-mg doses than with the 1,500-mg single dose. The accumulation ratios for *C*_max_ and AUC from time zero to the 6- or 12-h dosing interval (AUC_0–τ_) were approximately 40% higher when 2 doses were administered 6 h apart than when they were administered 12 h apart. Mean *t*_1/2_, apparent clearance (CL/*F*), and apparent volume of distribution (*V_z_*/*F*) values were similar across the dose regimens.

**FIG 5:**
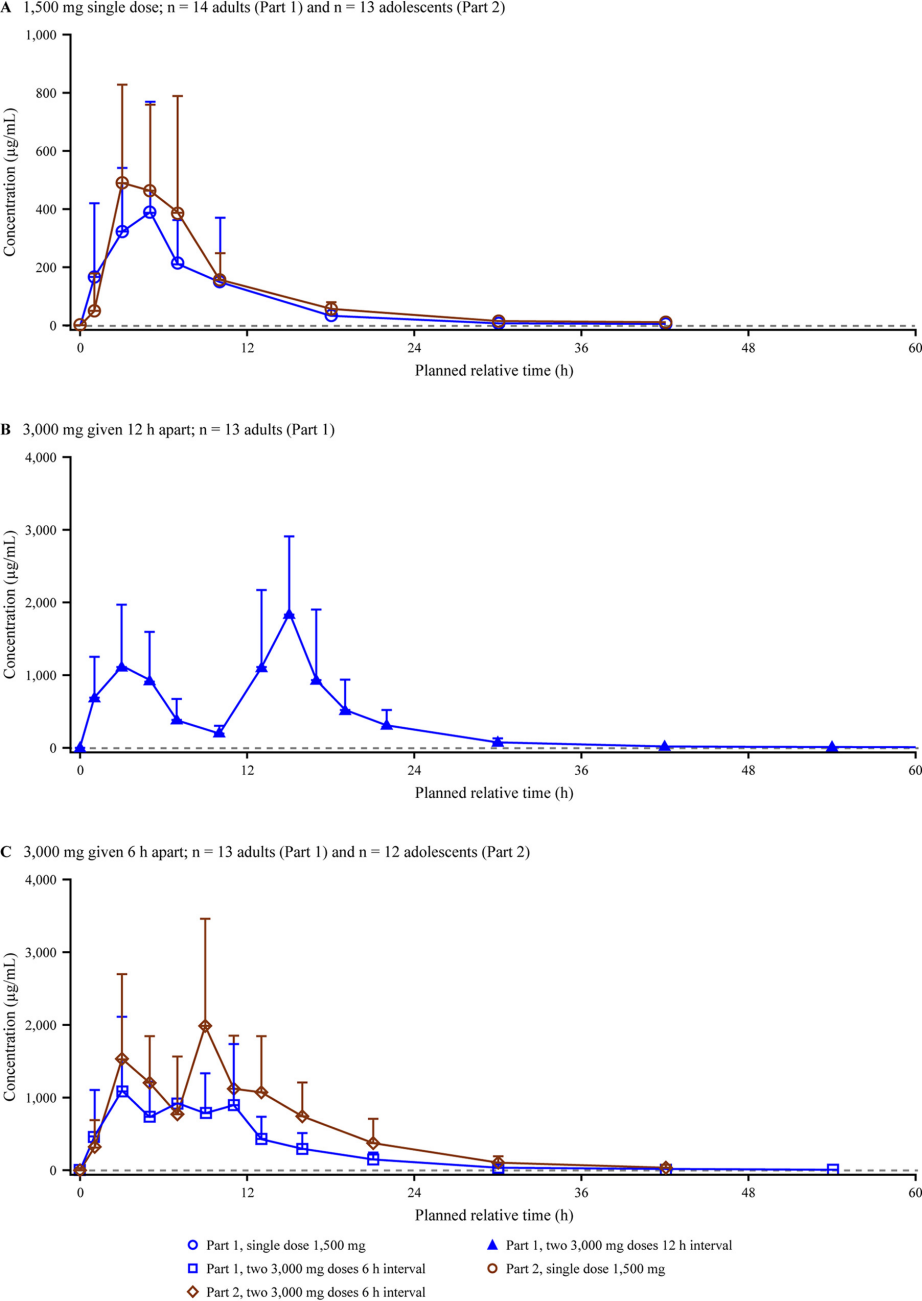
Arithmetic mean (SD) gepotidacin urine concentration-time plots on a linear scale for the adult and adolescent study. Note that study part 1 included adults and study part 2 included adolescents. The lower limit of quantification is 1 μg/mL, represented by the dashed line. As urine collection is representative of a time interval and not a single time point, the average concentrations at the midpoint of the collection intervals are used for plotting purposes only.

Urine gepotidacin AUCs from time zero to 48 h postdose (AUC_0–48_) were 4- and 5.5-fold higher after two 3,000-mg doses 6 h apart and 12 h apart, respectively, than with the 1,500-mg single dose (Fig. 5 and Table 3). As urine collection is representative of a time interval and not a single time point, the average concentrations at the midpoint of the collection intervals are used for plotting purposes in Fig. 5, while Table 3 details the specific time point from time zero to the time point indicated. Renal clearance and *fe*% were similar across the dose regimens.

### (ii) Adolescent pharmacokinetics.

Gepotidacin plasma concentrations peaked 3.00 h after single-dose administration. When 2 doses of gepotidacin were administered 6 h apart, plasma concentrations peaked 2.75 h after the first dose and 1.50 h after the second dose (Fig. 4). Maximum plasma concentrations were approximately 2.4-fold higher after the first 3,000-mg dose than with the 1,500-mg single dose, and the AUC_0–_*_t_* was approximately 5-fold higher after two 3,000-mg doses given 6 h apart than with the 1,500-mg single dose (Table 2). The accumulation ratios for AUC_0–τ_ and *C*_max_ were 1.66 and 1.32, respectively, for the 6-h dosing interval. The *t*_1/2_ estimates and *V_z_*/*F* were higher for the single dose than for 2 doses 6 h apart; however, CL/*F* estimates were similar for both dose regimens.

The gepotidacin urine AUC_0–48_ was 5.3-fold higher after two 3,000-mg doses 6 h apart than with the single 1,500-mg dose, with similar CL_R_ and *fe*% values across the dose regimens (Fig. 5 and Table 3).

### (iii) Pharmacokinetic comparisons.

**FIG 6:**
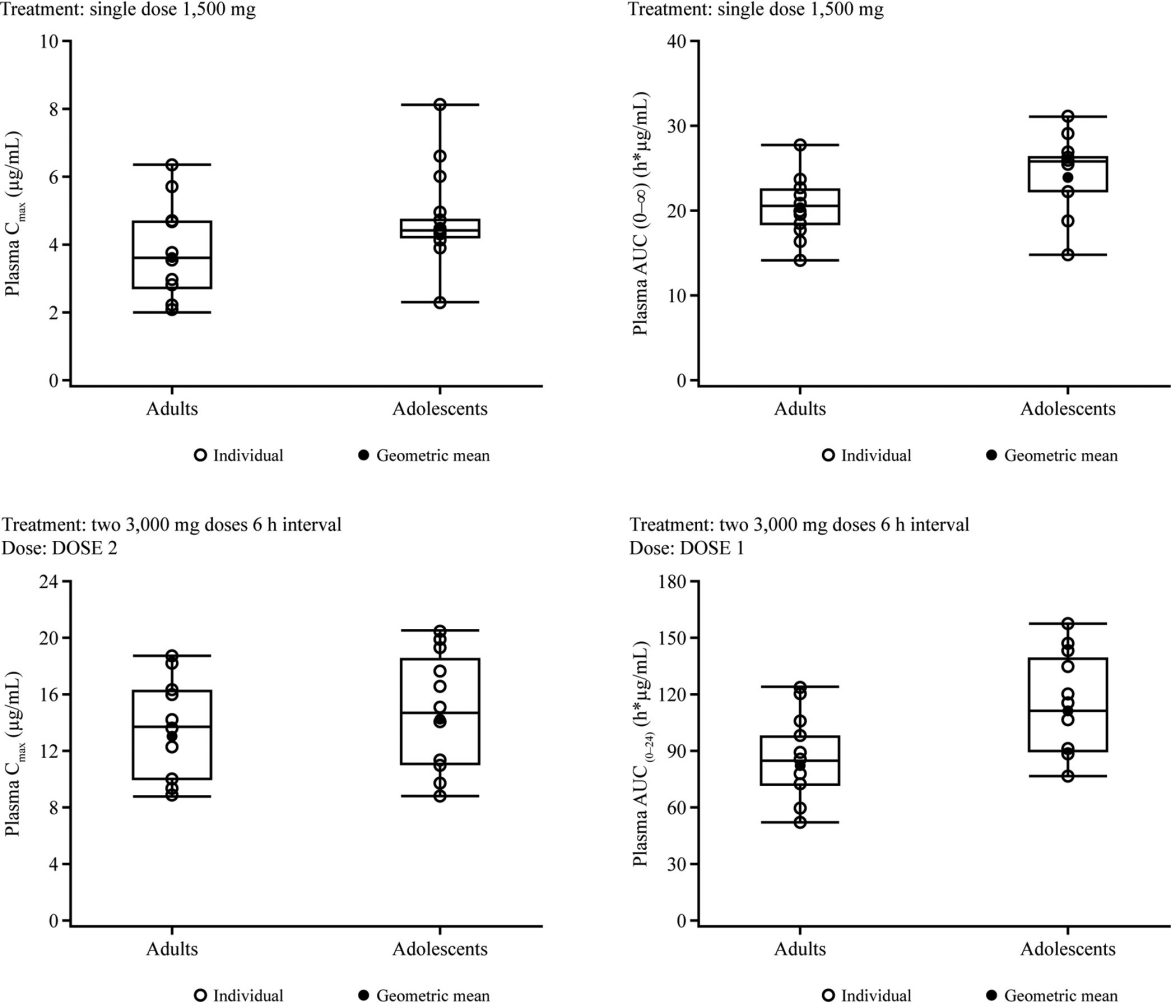
Box-and-whisker plots of plasma gepotidacin *C*_max_ and AUC after a single 1,500-mg dose (*n* = 14 adults, and *n* = 13 adolescents) and 3,000 mg given 6 h apart (*n* = 13 adults, and *n* = 12 adolescents) in the adult and adolescent study. Note that the bottom and top box lines represent the lower and upper quartiles, Q1 and Q3, respectively, with the median represented by the middle box line. Top and bottom whiskers present the maximum and minimum values, respectively. The mean is represented by a closed circle. Individual data are presented as open circles. For the two 3,000-mg doses, *C*_max_ is presented for dose 2, and the AUC_0–24_ was estimated using the full profile.

After the single 1,500-mg dose, mean *C*_max_ values were 27% higher in adolescents than in adults, with similar *C*_max_ ranges and comparable AUC_0–∞_ values in both populations (Fig. 6 and Table 2). Likewise, after the first of two 3,000-mg doses was administered 6 h apart, the mean *C*_max_ value was 29% higher in adolescents than in adults, with similar *C*_max_ ranges in both populations. Overall, *C*_max_ ranges and mean *C*_max_ values were similar in adults and adolescents after the second 3,000-mg dose. The mean AUC from time zero to 24 h (AUC_0–24_) was approximately 35% higher in adolescents after the administration of 2 doses 6 h apart than in adults. The mean *t*_1/2_ values were similar in both populations after the single dose, with values of 11.5 h and 13 h in adults and adolescents, respectively. After 2 doses 6 h apart, the mean *t*_1/2_ in adolescents was notably lower (6.98 h) than that in adults (12 h).

There were no differences observed in the total amount of gepotidacin excreted in urine between adults and adolescents after a single dose; however, after 2 doses 6 h apart, the total amount excreted was approximately 35% higher in adolescents than in adults (Table 3).

### (iv) Effect of emesis on pharmacokinetic data.

Seven participants (2 adults and 5 adolescents) experienced emesis when gepotidacin was administered as 2 doses given 6 or 12 h apart (Table S2), whereas no participants had emesis after the single dose of gepotidacin. Of these 7 participants, 1 adult experienced emesis after both dosing regimens of two 3,000-mg doses 6 and 12 h apart. The remaining 6 participants experienced emesis only after two 3,000-mg doses 6 h apart; the majority had emesis onset after the second dose. The time of onset of emesis ranged from approximately 1 to 4.5 h; there were no acute events of emesis that occurred less than 1 h after dosing (Table S2).

The data from these participants were not excluded from the analysis; however, the PK data were investigated for any impact of emesis. The plasma concentration-time profiles and the PK parameter values for these participants were generally similar or were within the range of values for participants without emesis, suggesting that emesis did not significantly impact gepotidacin exposure (Table S3 and Fig. S1).

### (v) Pharmacokinetics and pharmacodynamics.

**FIG 7:**
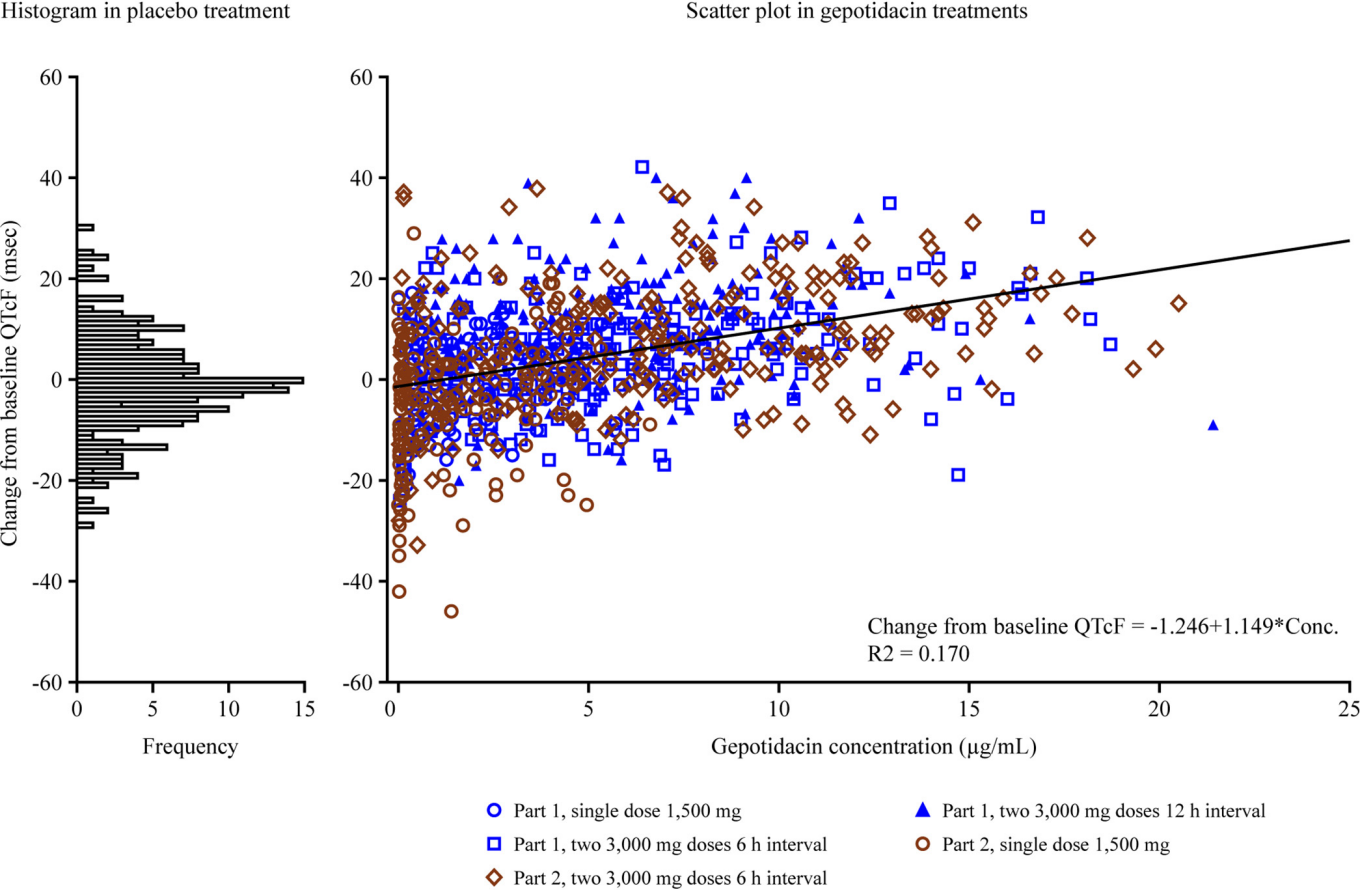
Change from baseline QTcF data versus gepotidacin plasma concentrations for adult and adolescent participants in the adult and adolescent study. Note that study part 1 included adults (*n* = 14 for a single dose of 1,500 mg, *n* = 13 for two 3,000-mg doses at a 6-h interval, and *n* = 13 for two 3,000-mg doses at a 12-h interval) and study part 2 included adolescents (*n* = 13 for a single dose of 1,500 mg, and *n* = 12 for two 3,000-mg doses at a 6-h interval). QTcF, corrected QT interval using the Fridericia formula.

The change from baseline in the corrected QT interval (QTc) for heart rate according to Fridericia (QTcF) data showed a slight positive trend of increasing as exposure to gepotidacin increased (Fig. 7). No individual adolescent or adult changes from baseline in QTcF were clinically meaningful, defined as a change from the baseline of >60 ms. No participants had a postdose QTcF value of >500 ms.

### (vi) Safety and tolerability.

**FIG 8:**
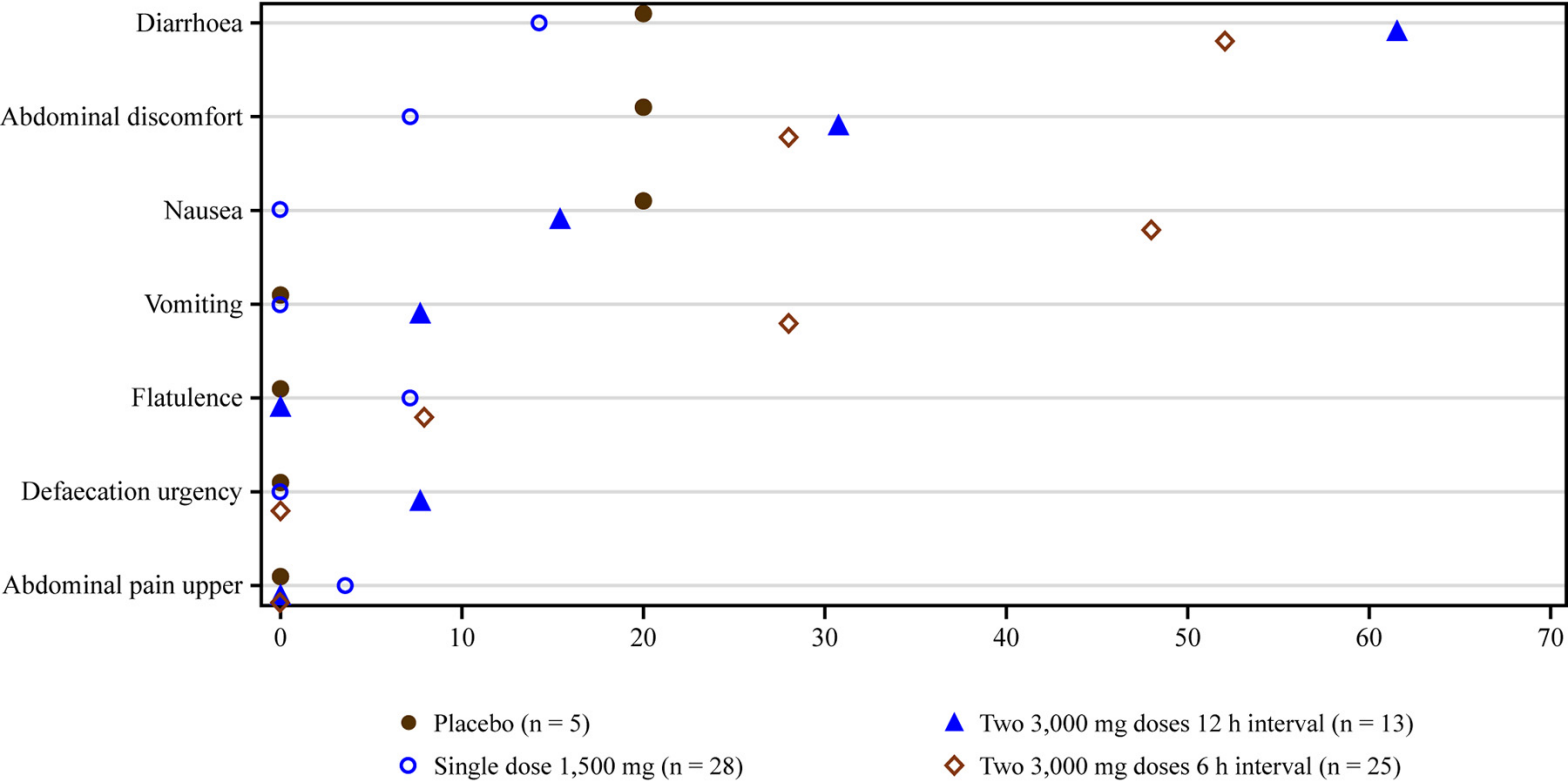
Plot of gastrointestinal adverse events of special interest in the adult and adolescent study. Note that data from both study part 1 and study part 2 are presented.

A total of 69% of adult and 88% of adolescent participants experienced AEs during the study (Tables 5 and 6). After receiving placebo, 2 adolescent participants and no adults experienced AEs. In both populations, the incidence of AEs was lowest after the 1,500-mg single dose and higher when two 3,000-mg doses were administered 6 or 12 h apart. For the single dose, 7% of adults and 64% of adolescents experienced AEs. After 2 doses 6 h apart, 69% and 100% of adults and adolescents experienced AEs, respectively. For 2 doses 12 h apart, which were administered only to adults, 77% of participants experienced AEs. In both populations, gastrointestinal AEs were the most prevalent, affecting 56% of adults and 82% of adolescents, and primarily included diarrhea, nausea, abdominal discomfort, and vomiting. More gastrointestinal events occurred after the two 3,000-mg doses; the increase in AEs as the dose increased was observed in both adults and adolescents (Fig. 8). AEs of dizziness and headache were reported in 29% and 24% of adolescent participants; however, these AEs were not reported in adult participants.

**TABLE 5:** Summary of adverse events for oral gepotidacin mesylate salt tablets in healthy adult participants in the adult and adolescent study^*a*^

System organ class, preferred term	No. (%) of participants				
	1,500-mg single dose (*n* = 14)	2 × 3,000-mg doses 12 h apart (*n* = 13)	2 × 3,000-mg doses 6 h apart (*n* = 13)	Placebo (*n* = 2)	Total (*n* = 16)
Any adverse event	1 (7)	10 (77)	9 (69)	0	11 (69)
Gastrointestinal disorders	1 (7)	9 (69)	8 (62)	0	9 (56)
* Diarrhea*	1 (7)	8 (62)	8 (62)	0	9 (56)
Abdominal discomfort	0	4 (31)	4 (31)	0	6 (38)
* Nausea*	0	2 (15)	3 (23)	0	4 (25)
* Vomiting*	0	1 (8)	2 (15)	0	2 (13)
Defecation urgency	0	1 (8)	0	0	1 (6)
Cardiac disorders	0	1 (8)	0	0	1 (6)
Arrhythmia	0	1 (8)	0	0	1 (6)
Investigations	0	0	1 (8)	0	1 (6)
Blood creatine phosphokinase increased	0	0	1 (8)	0	1 (6)
Skin and subcutaneous disorders	0	0	1 (8)	0	1 (6)
Dermatitis	0	0	1 (8)	0	1 (6)

a The gepotidacin strength of the tablets administered was 750 mg; multiple capsules/tablets were administered to provide each required dose. Adverse event preferred terms presented in italics were associated with acetylcholinesterase inhibition in addition to the system organ class shown.

**TABLE 6:** Summary of adverse events for oral gepotidacin mesylate salt tablets in healthy adolescent participants in the adult and adolescent study^*a*^

System organ class, preferred term	No. (%) of participants			
	1,500-mg single dose (*n* = 14)	2 × 3,000-mg doses 6 h apart (*n* = 12)	Placebo (*n* = 3)	Total (*n* = 17)
Any adverse event	9 (64)	12 (100)	2 (67)	15 (88)
Gastrointestinal disorders	6 (43)	11 (92)	2 (67)	14 (82)
* Nausea*	0	9 (75)	1 (33)	10 (59)
* Diarrhea*	3 (21)	5 (42)	1 (33)	7 (41)
Abdominal discomfort	2 (14)	3 (25)	1 (33)	5 (29)
* Vomiting*	0	5 (42)	0	5 (29)
Flatulence	2 (14)	2 (17)	0	2 (12)
Upper abdominal pain	1 (7)	0	0	1 (6)
Nervous system disorders	2 (14)	4 (33)	1 (33)	6 (35)
Dizziness	0	4 (33)	1 (33)	5 (29)
Headache	2 (14)	1 (8)	1 (33)	4 (24)
Syncope	0	0	1 (33)	1 (6)
Cardiac disorders	1 (7)	1 (8)	0	2 (12)
Supraventricular extrasystoles	0	1 (8)	0	1 (6)
Tachycardia	1 (7)	0	0	1 (6)
General disorders and administration site conditions	0	1 (8)	0	1 (6)
Chest discomfort	0	1 (8)	0	1 (6)
Skin and subcutaneous disorders	1 (7)	0	0	1 (6)
Dermatitis contact	1 (7)	0	0	1 (6)

a The gepotidacin strength of the tablets administered was 750 mg; multiple capsules/tablets were administered to provide each required dose. Adverse event preferred terms presented in italics were associated with acetylcholinesterase inhibition in addition to the system organ class shown.

Across both populations, AEs were of mild intensity, with the exception of 6 participants who experienced AEs of moderate severity, as follows: 1 adult and 4 adolescents experienced vomiting after receiving gepotidacin, and 1 adolescent experienced syncope after receiving placebo. There were no deaths, serious AEs, or AEs that led to discontinuation.

**FIG 9:**
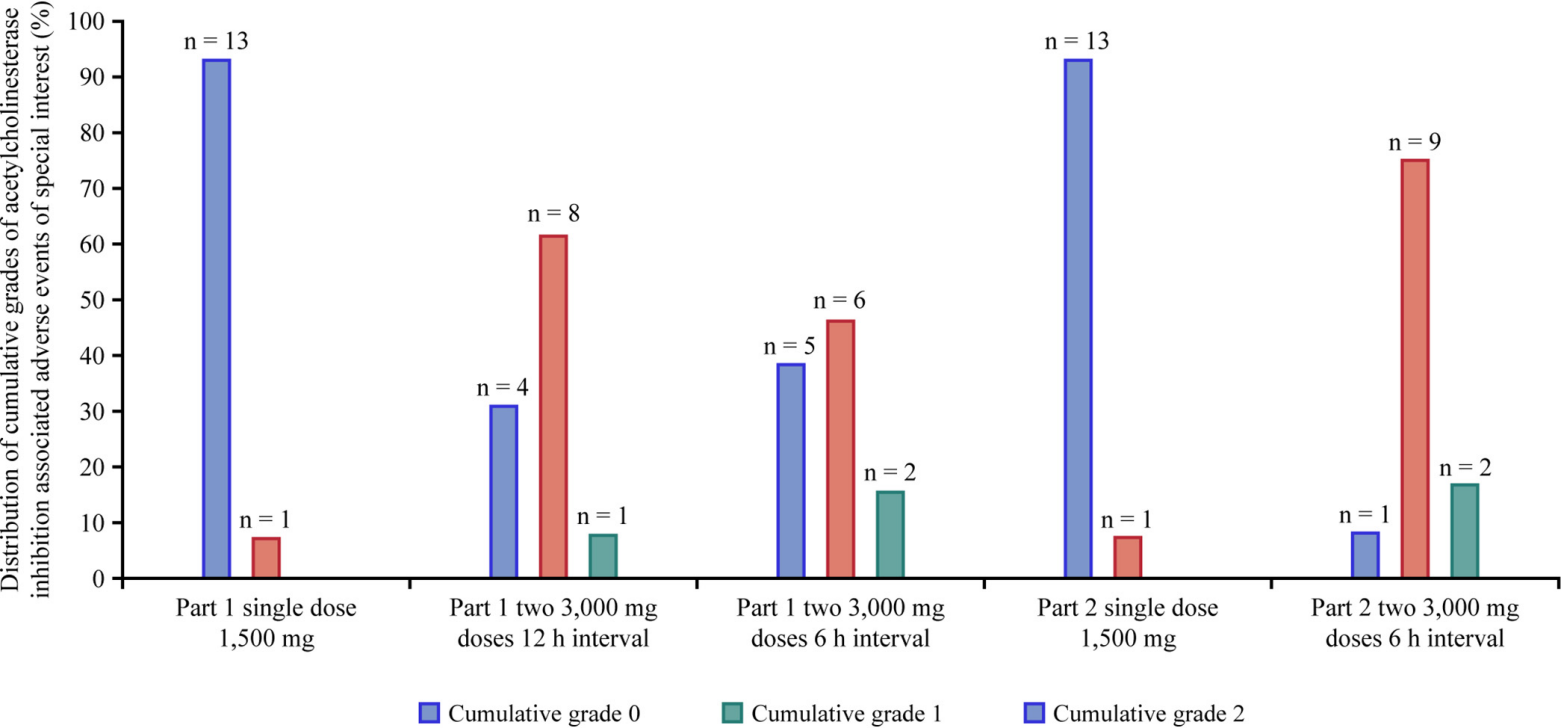
Percent distribution of cumulative grades of acetylcholinesterase inhibition-associated adverse events of special interest in the adult and adolescent study. Note that data from both study part 1 and study part 2 are presented.

Based on the known safety profile for oral gepotidacin, AEs of special interest (AESIs) were specifically assessed in the analysis for cardiovascular and gastrointestinal events and events potentially related to acetylcholinesterase inhibition. A total of 3 participants across both populations had cardiovascular AESIs, all were mild, and 1 AE of supraventricular extrasystoles was considered related to gepotidacin by the investigator. Gastrointestinal AESIs were experienced by 9 adult participants and 14 adolescent participants; the most commonly reported AE was diarrhea in each population. Events compatible with acetylcholinesterase inhibition were observed in 9 adults and 13 adolescents and had a notable overlap with gastrointestinal events of nausea, diarrhea, abdominal discomfort, vomiting, and defecation urgency (Tables 5 and 6). Cumulative grades for acetylcholinesterase inhibition-related AESIs increased as the gepotidacin dose increased from a 1,500-mg single dose to two 3,000-mg doses 6 or 12 h apart (Fig. 9).

No clinically significant trends were observed in laboratory values, ECG parameters, or vital sign measurements in either population. No Holter abnormalities were identified throughout the study.

## DISCUSSION

Throughout the clinical development program for gepotidacin, several different formulations have been evaluated. Initial clinical studies were conducted using gepotidacin mesylate salt capsule and free-base intravenous formulations. With progression to phase 2 evaluations and the identification of gepotidacin dose levels needed for efficacy, immediate-release film-coated tablets were developed to offer an acceptable size for patient convenience, to support a lower number of tablets per dose for patient compliance, and for manufacturing and scale-up feasibility purposes.

The first gepotidacin tablet formulation developed was a 750-mg-strength tablet using mesylate salt, which was compared in a relative bioavailability study to the reference 500-mg-strength capsule using mesylate salt at a total dose level of 1,500 mg under fed and fasted conditions (19). Both the tablet and reference capsule resulted in the rapid absorption of gepotidacin, with a median *T*_max_ of 1.5 to 1.75 h under fasted conditions. The bioavailability of the tablet was comparable to that of the capsule under fasted conditions at a 1,500-mg dose, with geometric least-squares mean ratios of the test tablet to the reference capsule (90% CIs) of 1.079 (1.019, 1.144) for AUC_0–∞_ and 1.036 (0.932, 1.152) for *C*_max_. Additionally, food was shown to have a minimal effect on the rate and extent of absorption of the tablet in healthy adult participants.

The relative bioavailability data presented here evaluated 2 different tablet formulations that were manufactured with gepotidacin as a free base, rather than mesylate salt. The free-base RC and HSWG tablets were assessed against the reference mesylate salt capsule. Although this study was not designed to assess bioequivalence, the 90% CIs of the AUC_0–∞_ and *C*_max_ parameter ratios for the RC tablet were within bioequivalence confidence bounds (18), and PK comparable to that of the reference capsule was also observed for the HSWG tablet, except for a slightly higher *C*_max_. After oral 1,500-mg gepotidacin dose administration via RC or HSWG tablets or reference capsules, gepotidacin urine concentrations of >4 μg/mL were achieved and maintained over 24 h postdose, which is supportive of the uncomplicated UTI indication (9).

Pharmacokinetic comparison of data from the first study using the mesylate salt tablet (19) with data from the current relative bioavailability study of free-base RC and HSWG tablets showed similar plasma exposures. Comparing PK parameters across studies, after single-dose administration of gepotidacin at 1,500 mg under fasted conditions, mean AUC_0–∞_ values were 15.8, 17.5, and 18.6 μg · h/mL and mean *C*_max_ values were 4.37, 4.49, and 5.35 μg/mL for the first mesylate salt tablet studied (19), the free-base RC tablet, and the free-base HSWG tablet, respectively. The mean *t*_1/2_ values were 11.8 h for the mesylate salt tablet and 10.2 h for both free-base tablets. Given adequate AUC and *C*_max_ exposures with the initial mesylate salt tablet formulation, and based on dissolution rates, a mesylate salt tablet formulation was further developed. A free-base 750-mg tablet formulation was evaluated in a phase 2a PK study in female participants with uncomplicated UTI (9), which demonstrated adequate systemic plasma and urine exposures, potential clinical and microbiological efficacy, and an acceptable safety-risk profile. This led to further formulation development and clinical evaluation of the 750-mg oral mesylate salt gepotidacin tablets in adults and adolescents as presented here.

The adult and adolescent study presented here was the first PK and safety clinical evaluation of the final, planned-to-be-commercial oral mesylate salt 750-mg tablet formulation for gepotidacin, not only in adults but also in an adolescent population. The data from the single 1,500-mg dose and the two 3,000-mg doses given 6 or 12 h apart supported the dose selections in the ongoing phase 3 studies in uncomplicated UTI (gepotidacin at 1,500 mg twice daily for 5 days [ClinicalTrials.gov identifiers NCT04020341 and NCT04187144]) and urogenital gonorrhea (gepotidacin at two 3,000-mg doses 10 to 12 h apart [ClinicalTrials.gov identifier NCT04010539]), respectively. Gepotidacin plasma AUC_0–∞_ values were similar across the age populations for the 1,500-mg single-dose administration, although *C*_max_ was found to be 27% higher in adolescents than in adults. With the administration of 2 doses, the plasma AUC_0–24_ was approximately 35% higher in adolescents than in adults after both the first and second doses; however, *C*_max_ was higher only after the first dose, by 29%, and was then found to be similar in both populations after the second dose. Thus, after the administration of 2 doses, plasma AUC_0–24_ but not *C*_max_ was elevated in adolescents. Assessments of these PK parameters are important; in a study of gepotidacin treatment of urogenital gonorrhea caused by Neisseria gonorrhoeae, AUC_0–24_ exposure was a driver for gepotidacin efficacy as it impacted the ratio of the AUC of the free, unbound fraction of the drug (*f*AUC)/MIC (20); also note that the *f*AUC_0–24_/MIC ratio has been identified as the index best describing the efficacy of gepotidacin in the treatment of uncomplicated UTI caused by Escherichia coli. In addition, increased *C*_max_ is related to a safety concern for QTc prolongation (15). A feasibility study to assess a modified-release formulation of gepotidacin to blunt the *C*_max_ and the magnitude of the QTc effect is planned. Overall, based on the PK and safety data profiles, the inclusion of adolescent participants (≥12 years of age) in the phase 3 studies who weigh ≥40 kg was supported. Of interest, the adolescent participants weighed approximately 15 kg less than the adult participants; a weak correlation between plasma PK parameters and body weight was observed and showed the lower the body weight, the higher the gepotidacin exposure (see Fig. S2 in the supplemental material). In addition, the mean *t*_1/2_ was approximately 5 h shorter in adolescents than in adults (6.98 h versus 12 h, respectively) when two 3,000-mg doses were administered 6 h apart (Table 2), but the terminal elimination profiles were similar, suggesting that the *t*_1/2_ values would be similar between populations (Fig. S3). Thus, this may be an artifact of the PK sample collection time points, where the last sample was collected 48 h after the second dose in adolescents; however, in adults, there was an additional 60-h-postdose collection, which may have impacted the slope determination for the elimination rate.

Several adult and adolescent participants experienced emesis shortly after gepotidacin administration; thus, the impact of emesis on the PK data was investigated (Tables S2 and S3 and Fig. S1). Of the 6 participants with emesis after receiving 2 doses 6 h apart only, the majority had emesis onset across a range of approximately 1.5 to 4.5 h after the second dose. The individual *T*_max_ values (Table S2) for these 6 participants do not suggest a consistent trend of occurrence before or after emesis. The *T*_max_ range for participants with emesis ranged from 1 to 3 h, which was within the *T*_max_ range for participants without emesis (0.50 to 5.42 h). No meaningful differences were observed for *C*_max_ and AUC values across participants with or without emesis, suggesting that this was not a local gastric effect and that gepotidacin had reached a point of systemic absorption in the intestines before emesis onset. Overall, the occurrence of emesis did not impact the systemic plasma exposures in adults or adolescents.

From a safety perspective, in the relative bioavailability study of the free-base RC and HSWG tablets, there were no notable safety concerns observed. With the exception of 4 of 26 participants who experienced AEs of moderate severity that included diarrhea or headache, all other AEs were of mild severity. One participant with a moderate AE of diarrhea had a 2-h delay in gepotidacin administration in a subsequent study period; however, the participant received the full planned dose and completed the study.

In the commercial tablet formulation PK study, both adult and adolescent participants experienced AESIs that were primarily associated with gastrointestinal and acetylcholinesterase inhibition events, which was expected based on the diversity and overlap of AEs associated with acetylcholinesterase inhibition. Such AEs cross over a range of body systems, including gastrointestinal, neurological, respiratory, and cardiovascular (see the supplemental material). These AESIs increased in prevalence and severity as the dose increased from a 1,500-mg single dose to two 3,000-mg doses given 6 or 12 h apart (6,000-mg total dose) (Fig. 8 and 9). The majority of AESIs were of mild severity, and none led to discontinuation from the study. Overall, similar AE profiles were observed between the dose regimens and age populations, even with higher PK exposures in adolescents. Although the incidence of AEs was numerically higher in adolescents than in adults, the nature and intensity were similar. The reasons for this higher incidence are only speculative but could be related to anxiety associated with participating in a clinical trial. Based on the known AE profile for gepotidacin (3–10, 16, 17), gastrointestinal AEs are commonly expected and were observed in both adults and adolescents in the present study, with an increase in such AEs as the dose increased. It is anticipated that a proportion of gastrointestinal AEs are associated with an off-target effect of gepotidacin, for example, acetylcholinesterase, which results in cholinergic effects either systemic or local to the gastrointestinal tract. However, further explanation would require additional research to explore the mechanism of gastrointestinal AEs associated with gepotidacin.

In a previous thorough QTc evaluation, no QTcF effect of clinical importance was observed with higher exposures to gepotidacin, particularly at exposures of >14 μg/mL (15). Based on results from the present study in adults and adolescents (Fig. 7), there was no clinically relevant QTcF effect (i.e., no values of >500 ms and no increase from the baseline of >60 ms) associated with the systemic exposures of gepotidacin after total doses of 6,000 mg (i.e., 3,000 mg given 6 or 12 h apart) in either age population. Across the dose regimens and age groups, the cardiovascular safety-risk profiles were similar.

In the adult and adolescent study, the only gepotidacin formulation evaluated was the 750-mg mesylate salt commercial tablet formulation; thus, this limits direct PK and safety comparisons to the previous mesylate salt capsules or capsules and the free-base tablets.

Overall, similar systemic plasma and urine exposures were observed after the administration of various tablet and capsule formulations of gepotidacin, including several observations within the 0.80-to-1.25 confidence bounds for plasma PK parameters. The PK profile for the commercial gepotidacin mesylate salt tablet formulation has been fully characterized. The data support including adolescents with a body weight of at least 40 kg in the phase 3 evaluations using the same adult doses and regimens. In addition, the dosing interval in the phase 3 pivotal study for urogenital gonorrhea was determined to be 10 to 12 h between the first and second doses to support adequate systemic concentrations while also minimizing *C*_max_ exposures. This increased time interval between doses may improve the gastrointestinal tolerability of the second dose by allowing an improvement or resolution of symptoms from the first dose while also minimizing *C*_max_-related adverse effects with the second dose. The urine exposures support the planned uncomplicated UTI indication from an MIC perspective (9). Data from both studies presented support an acceptable risk-safety profile for gepotidacin. The safety profile for the commercial gepotidacin tablet formulation in adult and adolescent participants was consistent with that observed in previous studies conducted in healthy participants, and no new safety concerns were identified. Ongoing phase 3 evaluations of the gepotidacin 750-mg mesylate salt tablet formulation will determine if this novel antibiotic will provide a much-needed oral therapeutic treatment option for uncomplicated UTI and urogenital gonorrhea.

## MATERIALS AND METHODS

The relative bioavailability (ClinicalTrials.gov identifier NCT02853435) and adult and adolescent (ClinicalTrials.gov identifier NCT04079790) studies were conducted in male or female (nonpregnant, nonlactating) participants in good health as determined by the investigator based on medical history, clinical laboratory results, vital sign measurements, 12-lead ECG results, and physical examination findings. Participants were between 18 and 64 years of age, inclusive, in the relative bioavailability study and the first part of the adult and adolescent study. In the second part of the latter study, adolescent participants were between 12 and 17 years of age, inclusive.

Both studies were conducted in accordance with the Declaration of Helsinki and Good Clinical Practice guidelines. The protocol and procedures were reviewed and approved by the IntegReview (Austin, TX, USA), Aspire (Santee, CA, USA), and Advarra (Columbia, MD, USA) institutional review boards. Written informed consent or assent was obtained from participants before any study procedures were performed.

### Relative bioavailability study. (i) Study design.

The relative bioavailability study was a 3-period crossover study to assess the relative bioavailability of a single 1,500-mg dose of gepotidacin in 2 free-base tablet formulations (two 750-mg RC and HSWG tablets) compared with the mesylate salt reference capsule formulation of gepotidacin (three 500-mg capsules). Each participant received all 3 treatments under fasted conditions according to an assigned treatment sequence based on a Latin-square design. There was a washout period of 3 days between doses. Participants were confined to the study site from period 1 through period 3 and returned to the study site for a follow-up visit 5 to 7 days after the last dose administration.

### (ii) Pharmacokinetic assessments.

Serial blood and urine samples were collected for PK analysis of gepotidacin concentrations up to approximately 48 h after each dose administration.

Blood samples were collected at predose and at 0.5, 1, 1.5, 2, 2.5, 3, 4, 6, 8, 12, 24, 36, and 48 h postdose. Urine was collected at predose and over the intervals of 0 to 2, 2 to 4, 4 to 6, 6 to 8, 8 to 12, 12 to 24, 24 to 36, and 36 to 48 h. The blood collection volume at each time point was approximately 3 mL.

### (iii) Safety assessments.

Throughout the study, AE monitoring, vital sign measurements, clinical laboratory evaluations, and physical examinations were performed. In addition, on-treatment ECGs were collected at 2, 24, and 48 h postdose and at the follow-up visit.

### Adult and adolescent study. (i) Study design.

The adult and adolescent study was a 2-part sequential study to evaluate the commercial gepotidacin 750-mg mesylate salt tablet formulation (Fig. 3). Part 1 was a 3-period, fixed-sequence assessment to evaluate the PK of a single 1,500-mg dose (period 1) and two 3,000-mg doses of gepotidacin administered 12 h apart (period 2) and 6 h apart (period 3) in healthy adult participants. Participants were randomly assigned to receive either active treatment (gepotidacin) or placebo for all 3 periods under fed conditions (a standard, moderate-fat meal). A total of 16 participants were randomized (in a 13:3 ratio) to receive a single dose of gepotidacin at 1,500 mg or matching placebo in period 1, gepotidacin at 3,000 mg given 12 h apart or matching placebo in period 2, and gepotidacin at 3,000 mg given 6 h apart or matching placebo in period 3. There was a washout period of 3 days between each period. Adult participants were confined to the study site from period 1 through period 3 and returned to the study site for a follow-up visit 4 to 10 days after the last dose administration.

Part 2 was a 2-period, fixed-sequence assessment to evaluate the PK of a single 1,500-mg dose (period 1) of gepotidacin and two 3,000-mg doses (period 2) of gepotidacin in healthy adolescent participants. The two 3,000-mg doses were administered 6 h apart; the time interval was determined based on PK and safety data from part 1. There was a washout period of 7 days between each period to accommodate school schedules. Participants were randomly assigned to receive either active treatment (gepotidacin) or placebo for both periods under fed conditions (a standard, moderate-fat meal). A total of 18 participants were randomized to receive a single dose of gepotidacin at 1,500 mg (*n* = 15) or matching placebo (*n* = 3) in period 1 and gepotidacin at 3,000 mg or matching placebo in period 2 to obtain 12 evaluable participants on active treatment and 2 evaluable participants on placebo. Adolescent participants were confined to the study site during each study period, were discharged after the 48-h assessments, and returned to the study site for a follow-up visit 4 to 10 days after the last dose administration.

### (ii) Pharmacokinetic assessments.

Serial blood and urine samples were collected for PK analysis of gepotidacin concentrations up to 48 h (adolescents) or 60 h (adults) after dose administration.

Blood samples were collected at predose and at 0.5, 1, 1.5, 2, 2.5, 3, 4, 6, 8, 12, 24, 36, and 48 h postdose for the single-dose administration in adults and adolescents. When gepotidacin was administered as 2 doses 12 h apart (adults only), blood samples were collected at predose and 0.5, 1, 1.5, 2, 2.5, 3, 4, 6, 8, 12, 12.5, 13, 13.5, 14, 14.5, 15, 16, 18, 20, 24, 36, 48, and 60 h after the first dose administration. When gepotidacin was administered as 2 doses 6 h apart (adults and adolescents), blood samples were collected at predose and 0.5, 1, 1.5, 2, 2.5, 3, 4, 6, 6.5, 7, 7.5, 8, 8.5, 9, 10, 12, 14, 18, 24, 36, and 48 h after the first dose administration; adults also had a 60-h-postdose blood sample collected. Blood collection volumes at each time point were approximately 3 mL in adults and 2 mL in adolescents.

Urine samples were collected at predose and over the intervals of 0 to 2, 2 to 4, 4 to 6, 6 to 8, 8 to 12, 12 to 24, 24 to 36, and 36 to 48 h for the single-dose administration in adults and adolescents. When gepotidacin was administered as 2 doses 12 h apart (adults only), urine samples were collected at predose and over the intervals of 0 to 2, 2 to 4, 4 to 6, 6 to 8, 8 to 12, 12 to 14, 14 to 16, 16 to 18, 18 to 20, 20 to 24, 24 to 36, 36 to 48, and 48 to 60 h. When gepotidacin was administered as 2 doses 6 h apart (adults and adolescents), urine samples were collected at predose and over the intervals of 0 to 2, 2 to 4, 4 to 6, 6 to 8, 8 to 10, 10 to 12, 12 to 14, 14 to 18, 18 to 24, 24 to 36, and 36 to 48 h; adults also had a 48- to 60-h-postdose collection.

### (iii) Safety assessments.

Throughout the study, AE monitoring, vital sign measurements, clinical laboratory evaluations, and physical examinations were performed. In addition, on-treatment ECGs time matched to the PK sampling time points were performed, and ECG Holter monitoring data were collected for 24 h after the first dose administration in each study period.

### Relative bioavailability and adult and adolescent studies. (i) Bioanalytical analysis.

All PK samples were analyzed using validated methods by PPD Bioanalytical Laboratory (Middleton, WI) to determine gepotidacin concentrations. Samples were analyzed by ultra- or high-performance liquid chromatography with tandem mass spectroscopy using positive ion electrospray validated over gepotidacin concentration ranges of 0.010 to 5.00 μg/mL for plasma and 1.00 to 500 μg/mL for urine.

### (ii) Analysis populations.

The analysis populations are defined in the supplemental material.

### (iii) Pharmacokinetic analysis.

Noncompartmental PK analyses were performed using Phoenix WinNonlin version 6.4 for the relative bioavailability study and Phoenix WinNonlin version 8 (Certara USA, Inc., Princeton, NJ) for the adult and adolescent study. Actual sampling times were used in both analyses. All data were based on total gepotidacin concentrations as plasma protein binding of gepotidacin is low (33%) (GlaxoSmithKline, unpublished data). Concentration-time data are presented as arithmetic means, and PK parameter data are presented as geometric means, unless otherwise indicated.

### (iv) Relative bioavailability analysis.

In the relative bioavailability study, the log-transformed AUC_0–_*_t_*, AUC_0–∞_, and *C*_max_ values for gepotidacin were analyzed separately using a mixed-effects model with fixed-effect terms for sequence, period, and regimen and with participant within sequence as a random effect. Point estimates and 90% CIs for the differences of interest (RC tablets and HSWG tablets versus reference mesylate salt capsules) were constructed using the residual variance. The *T*_max_ was analyzed nonparametrically using the Wilcoxon signed-rank test to compute the point estimate and 90% CI for the median difference for each comparison of interest. An analysis of variance was performed to obtain the geometric mean ratios between RC tablets and HSWG tablets versus reference mesylate salt capsules and 90% CIs for the ratios of AUC_0–_*_t_*, AUC_0–∞_, and *C*_max_.

### (v) Pharmacokinetic and pharmacodynamic QTcF analysis.

In the adult and adolescent study, QTcF evaluations and changes from baseline plots by gepotidacin concentrations were performed using SAS version 9.4 (SAS Institute, Inc., Cary, NC).

### (vi) Safety analysis.

Adverse events and changes from baseline values for clinical chemistry, hematology, vital signs, and ECG findings were analyzed using SAS version 9.3 for the relative bioavailability study and SAS version 9.4 (SAS Institute, Inc., Cary, NC) for the adult and adolescent study. For the adult and adolescent study, additional safety analyses were conducted to assess cardiovascular, gastrointestinal, and acetylcholinesterase inhibition-related AESIs. As acetylcholinesterase inhibition can be identified through multiple AEs, including an overlap with gastrointestinal events, 13 AE terms were identified in the analysis plan and via programming, and cumulative grades were determined (further analysis details are provided in the supplemental material).

### (vii) Interim data review.

In the adult and adolescent study, an interim data review was performed that consisted of reviewing data management listings and WinNonlin outputs. Preliminary plasma PK and safety results from adult participants in part 1 were reviewed by the sponsor and investigator before enrolling part 2 to determine the time interval between the 2 gepotidacin doses and to confirm PK sampling time points for adolescent participants.

### Data availability.

Anonymized individual participant data and study documents can be requested for further research from www.clinicalstudydatarequest.com.
