# Supplementary material for: Pharmacokinetics of Oral Formulations of Gepotidacin (GSK2140944), a Triazaacenaphthylene Bacterial Type II Topoisomerase Inhibitor, in Healthy Adult and Adolescent Participants
Source: Antimicrob Agents Chemother. 2022 Jan 18;66(1):e01263-21. doi: 10.1128/AAC.01263-21 (PMC8765319; doi:10.1128/AAC.01263-21)
Supplement: Supplemental file 1 — Supplemental material. Download AAC.01263-21-s0001.pdf, PDF file, 0.5 MB [file aac.01263-21-s0001.pdf]

Supplementary Materials

Pharmacokinetics of Oral Formulations of Gepotidacin (GSK2140944), a Triazaacenaphthylene Bacterial Type II Topoisomerase Inhibitor, in Healthy Adult and Adolescent Participants

Aline Barth, Mohammad Hossain, Darin B. Brimhall, Caroline R. Perry, Courtney A. Tiffany, Sherry Xu, Etienne F. Dumont

Supplementary Contents:

Relative Bioavailability Study – Analysis Populations

Adult and Adolescent Study – Analysis Populations

Acetylcholinesterase Inhibition Adverse Events of Special Interest – Analysis

Table 1. Relative Bioavailability Study – Analysis of Oral Gepotidacin Plasma Pharmacokinetic Parameters in Healthy Participants

Table 2. Adult and Adolescent Study – Emesis Time of Event Relative to Dose Administration and T_{max} Values

Table 3. Adult and Adolescent Study – Summary of Plasma Pharmacokinetic Parameters for Oral Gepotidacin in Participants With and Without Emesis

Figure 1. Adult and Adolescent Study – Individual Gepotidacin Plasma Concentration Time Plots by Treatment for Participants With and Without Emesis on Linear and Semilogarithmic Scales

Figure 2. Adult and Adolescent Study – Scatter Plots of Gepotidacin Body-Weight Adjusted AUC and C_{max} Plasma Pharmacokinetic Parameters on Linear Scale

Figure 3. Adult and Adolescent Study – Arithmetic Mean Gepotidacin Plasma Concentration Time Plots by Treatment on Semilogarithmic Scale

Relative Bioavailability Study – Analysis Populations

Population	Definition
Safety	Participants who received at least 1 dose of study drug and had at least 1 postdose safety assessment
PK	Participants who received at least 1 dose of gepotidacin and had evaluable PK data for gepotidacin
PK Parameter	Participants in the PK Population for whom valid and evaluable PK parameters were derived

Adult and Adolescent Study – Analysis Populations

Population	Definition
Randomized	All participants who were randomized
Safety	All participants who received at least 1 dose of gepotidacin
PK	All participants who received at least 1 dose of gepotidacin and had evaluable postdose plasma concentration data for gepotidacin
PK Parameter	All participants in the PK Population who received gepotidacin for whom valid and evaluable plasma PK parameters were derived for gepotidacin

Acetylcholinesterase Inhibition Adverse Events of Special Interest – Analysis

Any reported adverse event (AE) listed below with a start time no later than 12 h after the last dose administered in each treatment period, as evaluated by the investigator as per the Division of Microbiology and Infectious Disease (DMID) Adult Toxicity Tables for Adverse Event Assessment, were programmatically identified as acetylcholinesterase inhibition related AEs of special interest (AESIs).

List of AEs for programming that were considered due to acetylcholinesterase inhibition:

Gastrointestinal
- Nausea
- Vomiting
- Diarrhea
- Gastrointestinal cramping and pain

Neurological
- Seizure/Convulsions
- Vasovagal syncope
- Salivation
- Lacrimation
- Diaphoresis/sweating

Respiratory
- Bronchospasm (acute)
- Dyspnea
- Bronchorrhea

Cardiovascular
- Bradycardia

A cumulative grade was determined for acetylcholinesterase inhibition AESIs, calculated by the sum of the grade of each reported event. This enabled both the number and severity of each acetylcholinesterase inhibition AESIs to be taken into account. For example, if 2 AEs in the list above were reported, one of Grade 1 and one of Grade 3 according to DMID, the total Grade of 4 would result in a Cumulative Grade 2 per the table below.

Cumulative Grading of Acetylcholinesterase Inhibition AESIs

	Cumulative Grade 1	Cumulative Grade 2	Cumulative Grade 3	Cumulative Grade 4
**Total Grade of Signs & Symptoms**	1 to 3	4 to 6	7 to 10	≥11

Supplementary Table 1. Relative Bioavailability Study – Analysis of Oral Gepotidacin Plasma Pharmacokinetic Parameters in Healthy Participants[a]

Parameter	Treatment[b]	LS Geometric Mean	LS Geometric Mean Ratio (Test/Reference)	90% CI of the LS Geometric Mean Ratio
AUC_{0-t} (μg.h/ml)	MS Capsule Reference	16.5	–	–
	FB RC Tablet	17.2	1.0408	(0.9792, 1.1062)
	HSWG	18.4	1.1138	(1.0479, 1.1838)
$AUC_{0-\infty}$ (μg.h/ml)	MS Capsule Reference	16.8	–	–
	FB RC Tablet	17.5	1.0417	(0.9809, 1.1063)
	HSWG	18.7	1.1108	(1.0459, 1.1797)
Cmax (μg/ml)	MS Capsule Reference	4.68	–	–
	FB RC Tablet	4.48	0.9586	(0.8440, 1.0888)
	HSWG	5.37	1.1487	(1.0113, 1.3047)
**Parameter**	**Treatment[b]**	**Median Difference**	**90% CI of the Median Difference**	**P-value**
Median T_{max} (h)	MS Capsule Reference	–	–	–
	FB RC Tablet	–0.233	(–0.267, 0.000)	0.309
	HSWG	–0.492	(–0.500, –0.250)	<0.001

[a]CI, confidence interval; FB, free base, HSWG, high shear wet granulation; LS, least squares; MS, mesylate salt; RC, roller compacter.

[b]All treatments were 1,500 mg single doses. The gepotidacin strength of all capsules and tablets administered was 750 mg; multiple capsule/tablets were administered to provide each required dose.

Note: A mixed effects model with treatment, sequence, period as fixed effects and participant within sequence as random effect was performed on the natural log-transformed parameters AUC_{0-t}, $AUC_{0-\infty}$, and C_{max}. For T_{max}, the median difference and 90% CI of the median difference are from Hodge-Lehmann estimate. The p-value is from Wilcoxon signed-rank test.

Supplementary Table 2. Adult and Adolescent Study – Emesis Time of Event Relative to Dose Administration and T_{max} values

Study Part/Population	Participant[b]	2 × 3000 mg doses[a] 12 h apart			2 × 3000 mg doses[a] 6 h apart		
		Dose 1	Dose 2	T_{max} (h)	Dose 1	Dose 2	T_{max} (h)
1/Adult	1	–	–	Not applicable	–	2 h 44 min	1.50
1/Adult	2	1 h 6 min	–	1.57	–	1 h 21 min	1.50
2/Adolescent	3	–	–	–	–	2 h 41 min[c]	1.50
2/Adolescent	4	–	–	–	–	4 h 30 min	3.00
2/Adolescent	5	–	–	–	–	1 h 22 min	1.02
2/Adolescent	6	–	–	–	2 h 38 min[d]	[d]	1.50
2/Adolescent	7	–	–	–	–	1 h 32 min	1.00

[a]The gepotidacin strength of all mesylate salt tablets administered was 750 mg; multiple tablets were administered to provide each required dose.
[b]Participant numbers have been deidentified.
[c]When emesis occurred intermittently, times are from the first event.
[d]Participant experienced emesis after both Dose 1 and Dose 2.

Supplementary Table 3. Adult and Adolescent Study – Summary of Plasma Pharmacokinetic Parameters for Oral Gepotidacin in Participants With and Without Emesis

PK Parameter[b]	2 × 3000 mg doses[a] 12 h apart Adults				2 × 3000 mg doses[a] 6 h apart Adults				2 × 3000 mg doses[a] 6 h apart Adolescents			
	Dose 1		Dose 2		Dose 1		Dose 2		Dose 1		Dose 2	
	Emesis	Without Emesis	Emesis	Without Emesis	Emesis	Without Emesis	Emesis	Without Emesis	Emesis	Without Emesis	Emesis	Without Emesis
n[c]	1[d]	12	1[d]	12	2[e]	11	2[e]	11	5[f]	7	5[g]	7
AUC_{0-24} (µg.h/mL)	76.1 (–)	84.1 (59.1, 113)	–	–	121 (119, 123)	76.9 (51.6, 106)	–	–	105 (87.7, 143)	116 (75.8, 157)	–	–
AUC_{0-48} (µg.h/mL)	82.2 (–)	91.3 (63.4, 124)	–	–	125 (123, 127)	80.9 (54.8, 109)	–	–	109 (92.9, 148)	121 (78.9, 164)	–	–
$AUC_{0-\tau}$ (µg.h/mL)	30.2 (–)	38.9 (25.9, 52.2)	45.7 (–)	44.3 (30.1, 61.4)	–	24.1 (13.5, 46.4)	58.0 (57.5, 58.5)	37.5 (26.3, 53.7)	35.1 (–)	32.1 (21.3, 39.8)	50.5 (38.6, 73.1)	56.4 (34.5, 74.7)
C_{max} (µg/mL)	9.90 (–)	9.94 (6.89, 15.3)	11.9 (–)	11.0 (7.98, 21.4)	–	8.42 (4.52, 16.4)	18.2 (18.1, 18.2)	12.3 (8.79, 18.7)	13.6 (–)	10.6 (5.57, 14.3)	14.4 (8.76, 19.9)	14.2 (9.70, 20.5)
T_{max} (h)	1.50 (–)	2.00 (1.00, 4.00)	1.57 (–)	2.03 (1.00, 4.00)	–	2.63 (0.50, 5.42)	1.50 (1.50, 1.50)	1.50 (1.00, 3.28)	2.50 (–)	3.00 (1.00, 4.00)	1.50 (1.00,3.00)	2.00 (1.50,3.00)
T_{lag} (h)	0.00 (–)	0.00 (0.00, 0.00)	–	–	–	0.00 (0.00, 0.00)			0.00 (0.00, 0.00)	0.00 (0.00, 0.50)	–	–

[a]The gepotidacin strength of all mesylate salt tablets administered was 750 mg; multiple tablets were administered to provide each required dose.

[b]Values are presented as geometric mean (minimum, maximum) except for T_{max} and T_{lag}, which are presented as median (minimum, maximum). As AUC_{0-24} and AUC_{0-48} were estimated using the full profile, these parameters were impacted when participants had emesis following the second dose. When emesis occurred following the first dose, all PK parameters for both doses were impacted.

[c]The n represents the overall number of participants with or without emesis in the treatment subgroup.

[d]For this participant, emesis occurred after Dose 1; therefore, parameters for both Dose 1 and Dose 2 were impacted.

[e]For these 2 participants, emesis occurred after Dose 2; therefore, Dose 1 AUC_{0-24} and AUC_{0-48} parameters were impacted; however, the $AUC_{0-\tau}$, C_{max}, T_{max}, and T_{lag} following Dose 1 were not impacted.

[f]For 1 of these 5 participants, emesis occurred after Dose 1; therefore, parameters for both Dose 1 and Dose 2 were impacted.

[g]For 4 of these 5 participants, emesis occurred after Dose 2; therefore, Dose 1 AUC_{0-24} and AUC_{0-48} parameters were impacted; however, $AUC_{0-\tau}$, C_{max}, T_{max}, and T_{lag} following Dose 1 were not impacted.

Supplementary Figure 1. Adult and Adolescent Study – Individual Gepotidacin Plasma Concentration Time Plots by Treatment for Participants With and Without Emesis on Linear and Semilogarithmic Scales

Study Part 1 – Adults, 2 × 3000 mg doses 12 h apart (n = 13)

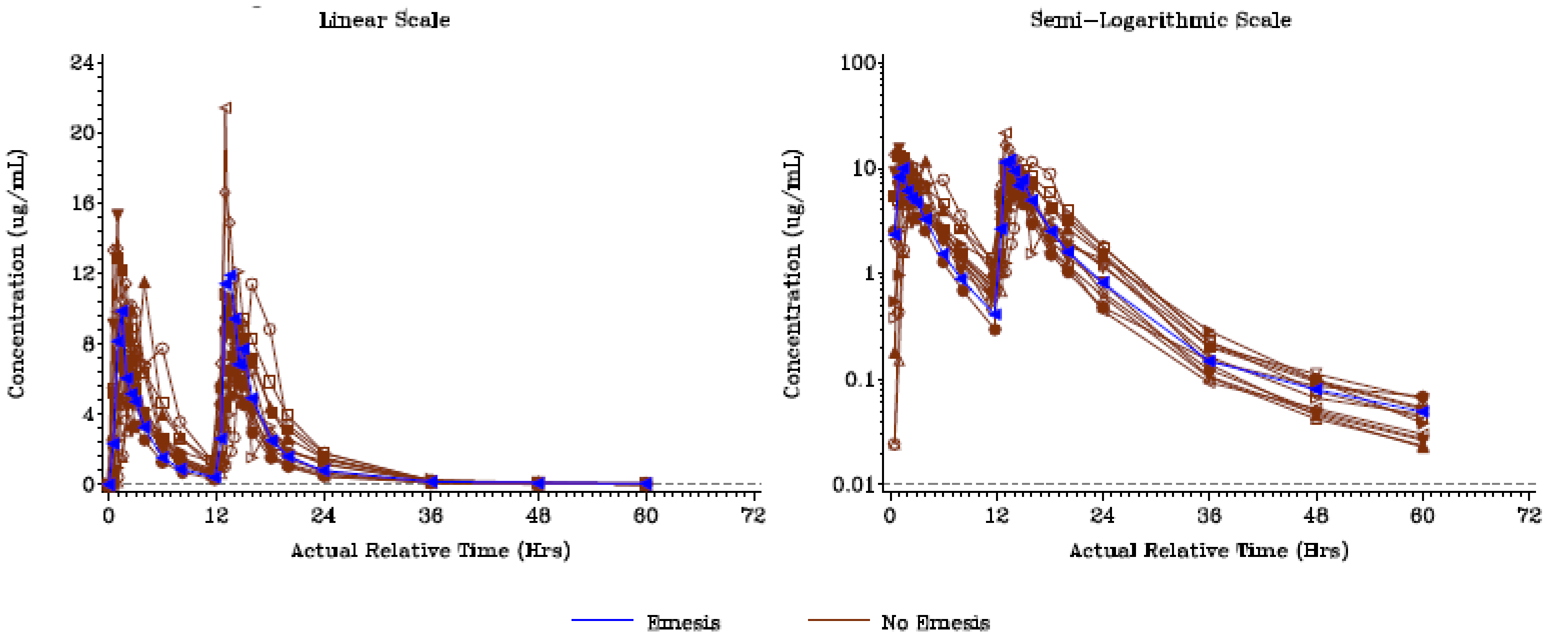

Study Part 1 – Adults, 2 × 3000 mg doses 6 h apart (n = 13)

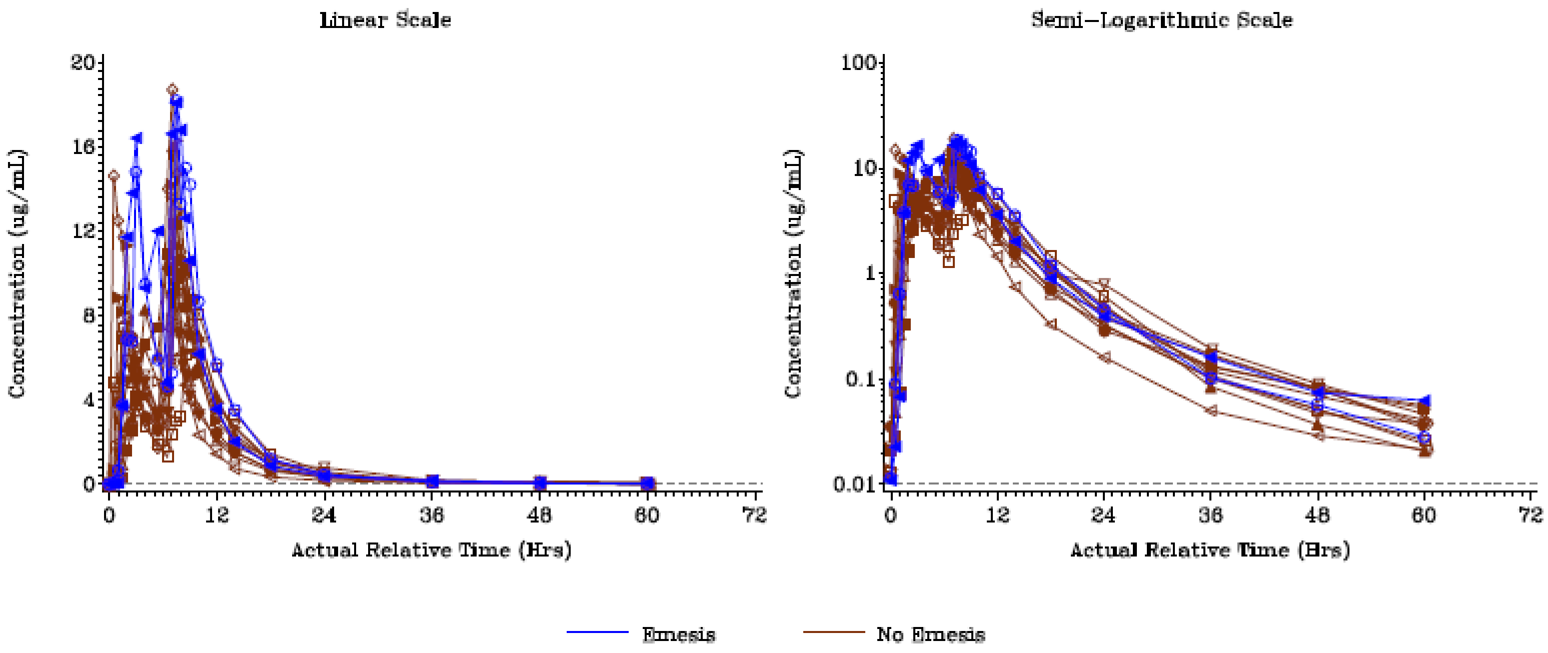

Study Part 2 – Adolescents, 2 × 3000 mg doses 6 h apart (n = 12)

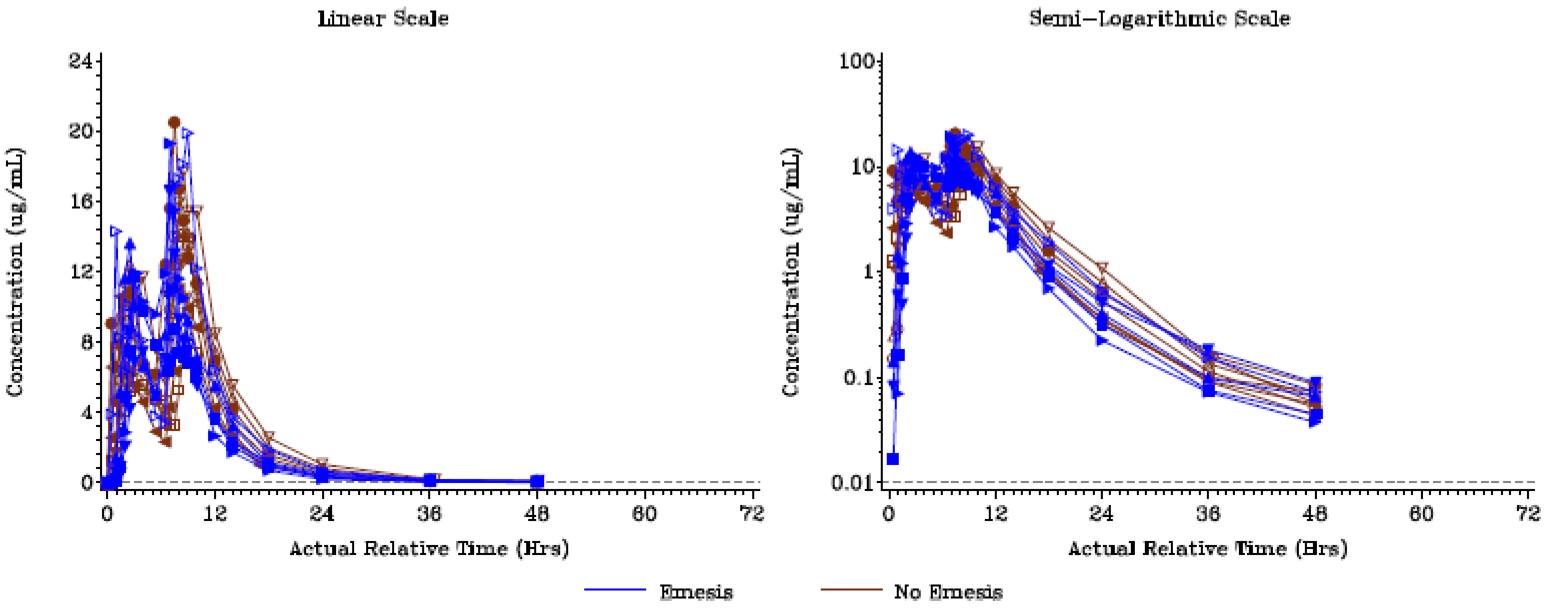

LLOQ = 0.0100 μg/mL. Dashed line represents LLQ.

Supplementary Figure 2. Adult and Adolescent Study – Scatter Plots of Gepotidacin Body-Weight Adjusted AUC and C_{max} Plasma Pharmacokinetic Parameters on Linear Scale

Study Part 1 – Adults (n = 14), Study Part 2 – Adolescents (n = 13), 1,500 mg single dose, $AUC_{0-\infty}$

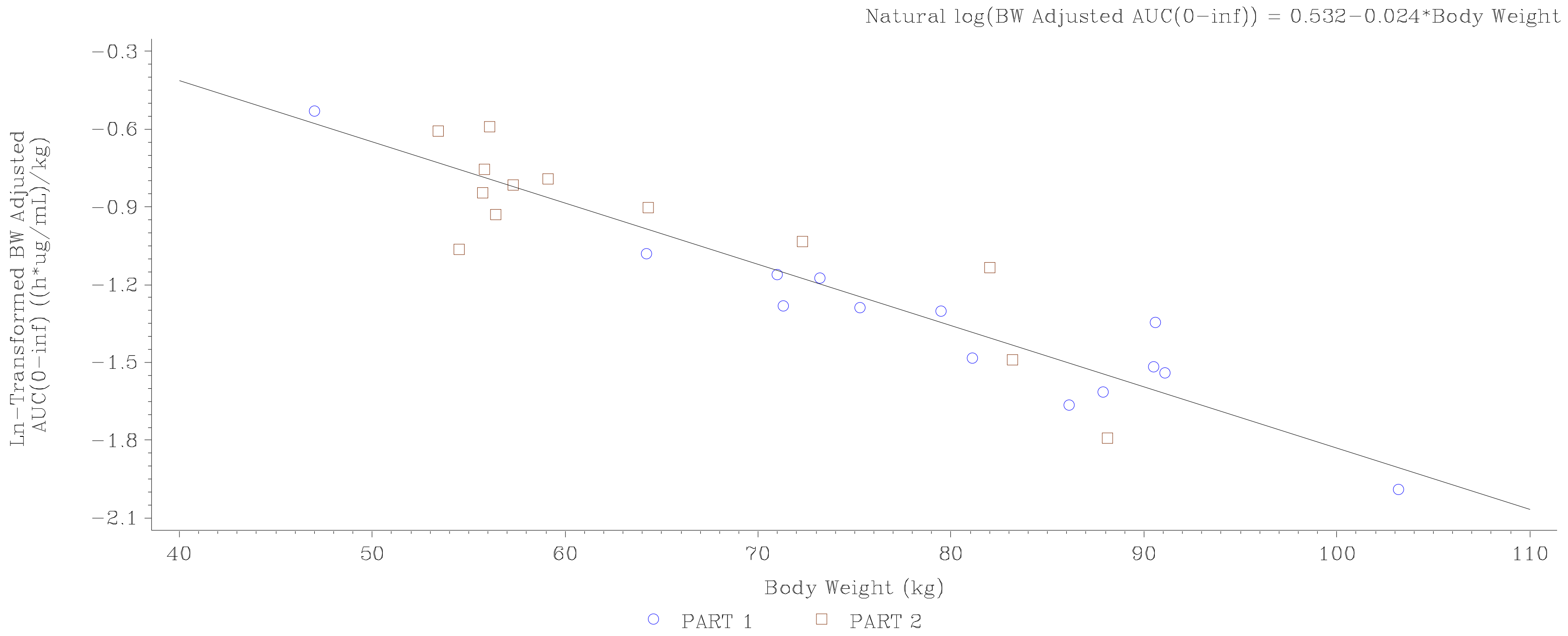

Study Part 1 – Adults (n = 14), Study Part 2 – Adolescents (n = 13), 1,500 mg single dose, C_{max}

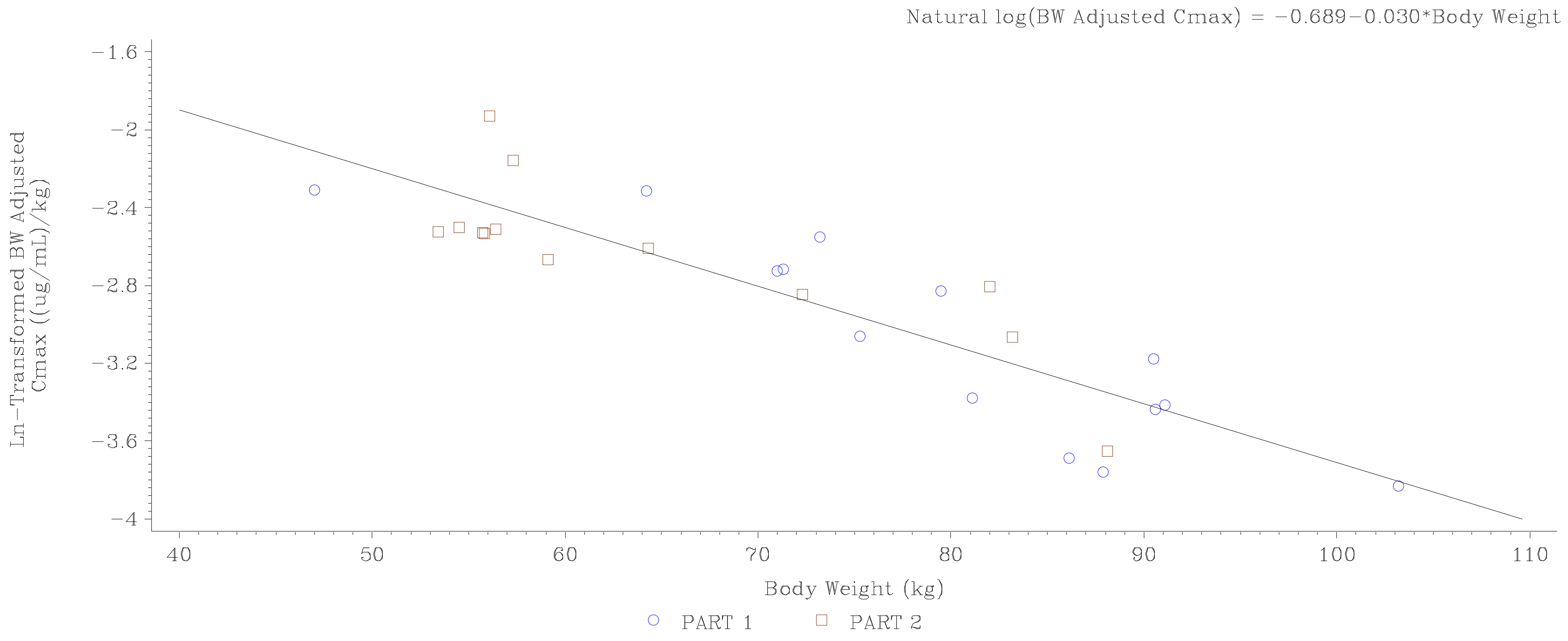

Study Part 1 – Adults (n = 13), Study Part 2 – Adolescents (n = 12), 2 × 3,000 mg doses 6 h apart, $AUC_{0\text{-}48}$

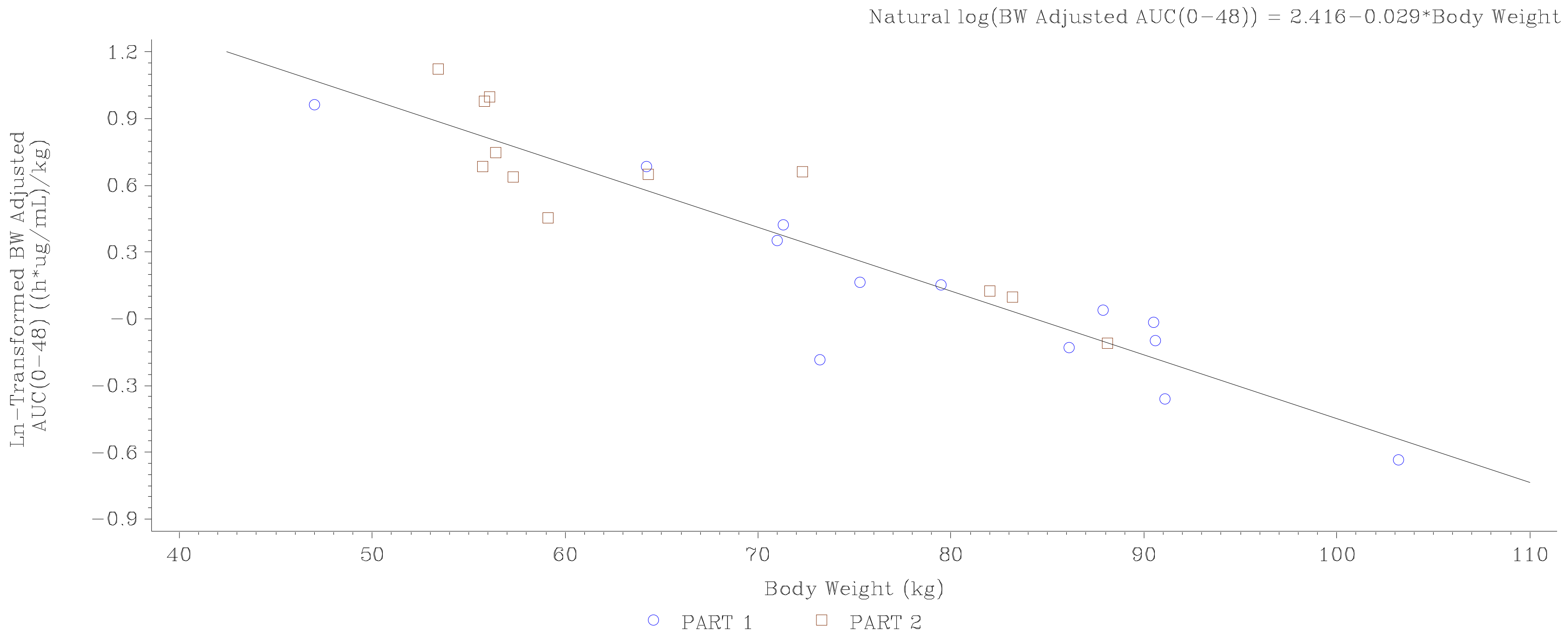

Study Part 1 – Adults (n = 13), Study Part 2 – Adolescents (n = 12), 2 × 3,000 mg doses 6 h apart, C_{max}

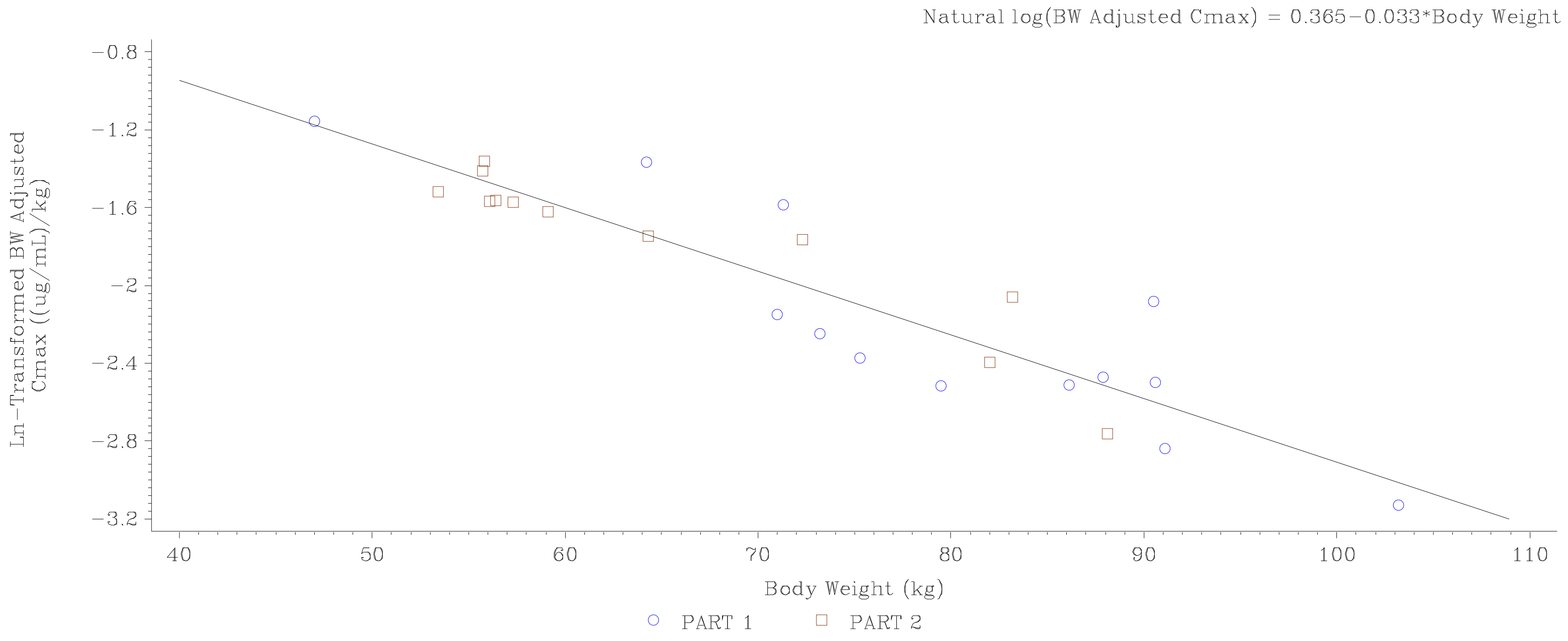

Supplementary Figure 3. Adult and Adolescent Study – Arithmetic Mean Gepotidacin Plasma Concentration Time Plots by Treatment on Semilogarithmic Scale

Single dose 1,500 mg (n = 14 adults and n = 13 adolescents)

2 × 3,000 mg doses 6 h apart (n = 13 adults and n = 12 adolescents)

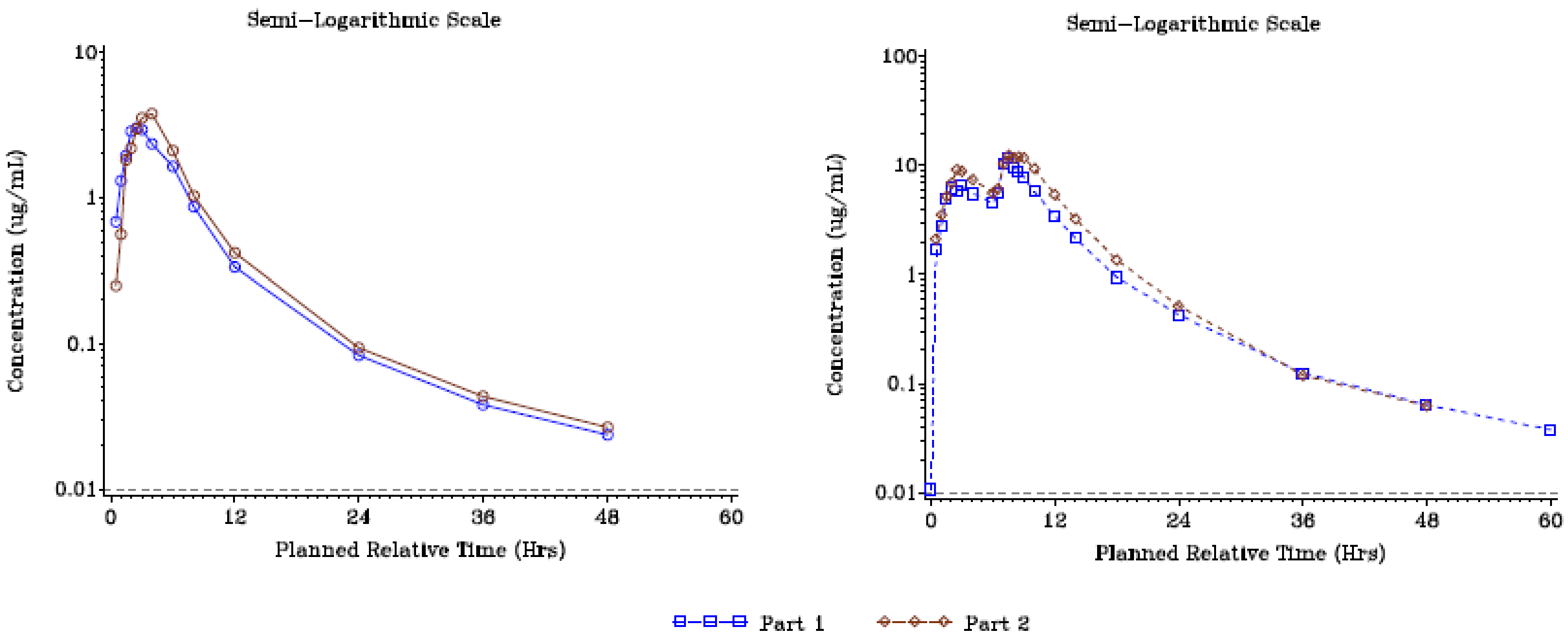

Study Part 1 – Adults; Study Part 2 - Adolescents
LLOQ = 0.0100 µg/mL. Dashed line represents LLQ.
